# Characterization of *CYP2D6* Pharmacogenetic Variation in Sub‐Saharan African Populations

**DOI:** 10.1002/cpt.2749

**Published:** 2022-10-21

**Authors:** David Twesigomwe, Britt I. Drögemöller, Galen E.B. Wright, Clement Adebamowo, Godfred Agongo, Palwendé R. Boua, Mogomotsi Matshaba, Maria Paximadis, Michèle Ramsay, Gustave Simo, Martin C. Simuunza, Caroline T. Tiemessen, Zané Lombard, Scott Hazelhurst

**Affiliations:** ^1^ Sydney Brenner Institute for Molecular Bioscience, Faculty of Health Sciences University of the Witwatersrand Johannesburg South Africa; ^2^ Division of Human Genetics, National Health Laboratory Service, and School of Pathology, Faculty of Health Sciences University of the Witwatersrand Johannesburg South Africa; ^3^ Department of Biochemistry and Medical Genetics, Rady Faculty of Health Sciences University of Manitoba Winnipeg Manitoba Canada; ^4^ Neuroscience Research Program, Kleysen Institute for Advanced Medicine, Winnipeg Health Sciences Centre and Max Rady College of Medicine University of Manitoba Winnipeg Manitoba Canada; ^5^ Department of Pharmacology and Therapeutics, Rady Faculty of Health Sciences University of Manitoba Winnipeg Manitoba Canada; ^6^ Institute for Human Virology Abuja Nigeria; ^7^ Division of Cancer Epidemiology, Department of Epidemiology and Public Health, and the Marlene and Stewart Greenebaum Comprehensive Cancer Centre University of Maryland School of Medicine Baltimore Maryland USA; ^8^ Navrongo Health Research Centre Ghana Health Service Navrongo Ghana; ^9^ C.K. Tedam University of Technology and Applied Sciences Navrongo Ghana; ^10^ Clinical Research Unit of Nanoro Institut de Recherche en Sciences de la Santé Nanoro Burkina Faso; ^11^ Botswana‐Baylor Children's Clinical Centre of Excellence Gaborone Botswana; ^12^ Retrovirology Department of Pediatrics, Baylor College of Medicine Houston Texas USA; ^13^ Centre for HIV and STIs, National Institute for Communicable Diseases, National Health Laboratory Services and Faculty of Health Sciences University of the Witwatersrand Johannesburg South Africa; ^14^ School of Molecular and Cell Biology University of the Witwatersrand Johannesburg South Africa; ^15^ Molecular Parasitology and Entomology Unit, Department of Biochemistry, Faculty of Science University of Dschang Dschang Cameroon; ^16^ Department of Disease Control, School of Veterinary Medicine University of Zambia Lusaka Zambia; ^17^ School of Electrical and Information Engineering University of the Witwatersrand Johannesburg South Africa

## Abstract

Cytochrome P450 2D6 (CYP2D6) is a key enzyme in drug response owing to its involvement in the metabolism of ~ 25% of clinically prescribed medications. The encoding *CYP2D6* gene is highly polymorphic, and many pharmacogenetics studies have been performed worldwide to investigate the distribution of *CYP2D6* star alleles (haplotypes); however, African populations have been relatively understudied to date. In this study, the distributions of *CYP2D6* star alleles and predicted drug metabolizer phenotypes—derived from activity scores—were examined across multiple sub‐Saharan African populations based on bioinformatics analysis of 961 high‐depth whole genome sequences. This was followed by characterization of novel star alleles and suballeles in a subset of the participants via targeted high‐fidelity Single‐Molecule Real‐Time resequencing (Pacific Biosciences). This study revealed varying frequencies of known *CYP2D6* alleles and predicted phenotypes across different African ethnolinguistic groups. Twenty‐seven novel *CYP2D6* star alleles were predicted computationally and two of them were further validated. This study highlights the importance of studying variation in key pharmacogenes such as *CYP2D6* in the African context to better understand population‐specific allele frequencies. This will aid in the development of better genotyping panels and star allele detection approaches with a view toward supporting effective implementation of precision medicine strategies in Africa and across the African diaspora.

**Study Highlights**

WHAT IS THE CURRENT KNOWLEDGE ON THE TOPIC?

*CYP2D6* genetic variation contributes to differences in cytochrome P450 2D6 (CYP2D6)–mediated drug metabolism across individuals and populations. *CYP2D6* is therefore an important gene in clinical pharmacogenetics implementation initiatives globally. Current catalogs of *CYP2D6* star alleles (haplotypes) are incomplete in part due to the high *CYP2D6* polymorphism and difficulty in interrogating the *CYP2D6* genomic locus.

WHAT QUESTION DID THIS STUDY ADDRESS?

The proportion of individuals with African ancestry across *CYP2D6* pharmacogenetics studies has been relatively low to date despite the known high genetic diversity in Africa. This study addresses the paucity of information about *CYP2D6* genetic variation across diverse African populations.

WHAT DOES THIS STUDY ADD TO OUR KNOWLEDGE?

This study highlights the largely nonuniform distributions of known and novel *CYP2D6* star alleles, and predicted metabolizer phenotypes across previously underrepresented African populations. We find that over 5% of the sub‐Saharan African participants carried potential novel *CYP2D6* star alleles. Predicted novel *CYP2D6* star alleles from our comparative analysis involving other global biogeographical groups are also provided. Furthermore, this study exemplifies the utility of high‐coverage whole genome sequence data and validated bioinformatics algorithms in catalyzing the investigation of haplotypes in hypervariable pharmacogenes such as *CYP2D6*.

HOW MIGHT THIS CHANGE CLINICAL PHARMACOLOGY OR TRANSLATIONAL SCIENCE?

This study highlights the need for clinical pharmacogenetics implementation across Africa and in global settings to optimize drug efficacy and safety, especially for commonly prescribed medications metabolized by CYP2D6. Our findings emphasize the fact that one African population should not be used as a general proxy for another—and certainly not for the whole of Africa or the African diaspora—when developing personalized medicine strategies.

Genetic variation is a major contributing factor to interindividual differences in drug response. Therefore, it is important to consider an individual's genetic profile in drug therapy (along with age, sex, environment, lifestyle, and other factors) in order to adjust treatment accordingly.^1^, ^2^, ^3^ This personalized medicine approach is aimed at promoting drug efficacy and minimizing the risk of adverse drug reactions. Individualizing treatment relies heavily on the ability to translate information about actionable variants and haplotypes into phenotype predictions, especially for key pharmacogenes involved in the absorption, distribution, metabolism, and excretion (ADME) of drugs. However, the distribution of known alleles in such genes varies considerably within and between populations.^4^, ^5^, ^6^ In addition, the full catalog of pharmacogenetic variants is yet to be ascertained, even for major pharmacogenes such as *CYP2D6*.^7^ This presents major challenges toward the application of current ADME genotyping panels and effective use of the activity score (AS) system for genotype–phenotype translation,^8^ especially in understudied populations.

CYP2D6 is a major ADME enzyme that has been widely studied given its involvement in the metabolism of 25% of clinically prescribed medications including opioids, antipsychotics, antidepressants, and anticancer agents (https://www.pharmgkb.org). The highly polymorphic *CYP2D6* gene is located on chromosome 22q13 alongside two homologous pseudogenes, *CYP2D7* and *CYP2D8*.^9^ The Pharmacogene Variation Consortium (PharmVar) has cataloged over 140 *CYP2D6* haplotypes (herein referred to as star alleles) to date (https://www.pharmvar.org). The functional impact of these star alleles ranges from no function to increased function as assigned by the Clinical Pharmacogenetics Implementation Consortium (CPIC) based on published experimental studies (https://cpicpgx.org). *CYP2D6* genotyping is complicated due to the occurrence of numerous combinations of star allele‐defining single‐nucleotide polymorphisms (SNPs) and indels (herein collectively referred to as SNVs) as well as structural variations.^10^ Star allele‐defining SNVs typically include missense, frameshift, splice‐site, stop‐gain, inframe deletion variants, and high‐impact regulatory region variants. SNVs that have no effect on the resulting protein sequence and/or function are typically included in “suballele” definitions by PharmVar. For example, CYP2D6*2 has over 25 suballeles (numbered from CYP2D6*2.001 onwards) due to the defining SNVs (rs16947 and rs1135840) occurring with various combinations of synonymous and benign intronic SNVs. Regarding structural variants (SVs), the *CYP2D6* copy number variations—gene deletion (CYP2D6*5) and gene duplication/multiplication alleles—are generally more common. However, other SVs such as hybrid gene copies comprising *CYP2D6* and *CYP2D7* portions have also been described. In particular, among East Asian populations, tandem rearrangements comprising CYP2D6*36 (exon 9 conversion to *CYP2D7*) and CYP2D6*10 are more common than typical copy number variations. *CYP2D6* SVs are notoriously difficult to investigate on currently available genotyping and/or targeted sequencing platforms.^11^, ^12^

At present, very few African ethnolinguistic groups have been represented in *CYP2D6* pharmacogenetics studies^5^ (see also https://www.pharmgkb.org/page/cyp2d6RefMaterials). This has been in part due to lack of sufficient high‐coverage genomic data sets, and also because of the genotyping challenges associated with the complex *CYP2D6* genomic locus.^13^ The most common African‐ancestry *CYP2D6* star alleles include the decreased‐function CYP2D6*17 and *29 alleles.^14^, ^15^ However, recent studies on more sub‐Saharan African cohorts have demonstrated unique *CYP2D6* star allele distributions and found novel, albeit relatively rare alleles. These rarer star alleles include CYP2D6*70, *73, *74, *84, and *106.^16^, ^17^, ^18^, ^19^ Some of the newer alleles, e.g., CYP2D6*73 (unknown function), *74 (unknown function) and *106 (uncertain function) represent new combinations of known potentially function‐altering variants on other existing haplotypes (https://www.pharmvar.org). Conversely, other alleles such as CYP2D6*84 were completely novel on discovery and submission to PharmVar. Furthermore, some star allele frequency differences are expected across Africa given that the population structure tends to correlate with geographical location as detailed by Choudhury *et al*.,^20^ da Rocha *et al*.,^21^ and Sengupta *et al*.^22^ This underscores the need for investigation of the *CYP2D6* allelic landscape across diverse African populations to reliably inform the design of comprehensive genotyping panels and the much‐needed clinical pharmacogenomics implementation strategies across Africa.

This study therefore aims to investigate the distribution of known and potential novel *CYP2D6* star alleles across diverse sub‐Saharan African (SSA) populations, and to predict the metabolizer phenotype distribution (based on diplotypes). In our analysis, we leverage the capabilities of recently developed and benchmarked star allele calling bioinformatics algorithms^23^, ^24^, ^25^, ^26^, ^27^ for determining *CYP2D6* diplotypes from short‐read high‐coverage whole genome sequence (WGS) data. In addition, we present the experimental validation of novel *CYP2D6* star alleles and suballeles identified in a subset of the African participants. The findings from this study have potential implications for clinical pharmacogenetics implementation strategies across Africa and in global settings with individuals of African ancestry.

## METHODS

### Ethics statement

This study was approved by the Human Research Ethics Committee (Medical) of the University of the Witwatersrand (Wits HREC‐Medical) (M200993). We performed secondary analysis of African WGS data that had been generated by other projects. This included data from public repositories and data from contributing Africa‐based studies/centers, each of which obtained additional local ethics approval (see **Supplementary Note** **S1**).

### Study population and whole genome sequence data sources

**Figure 1:**
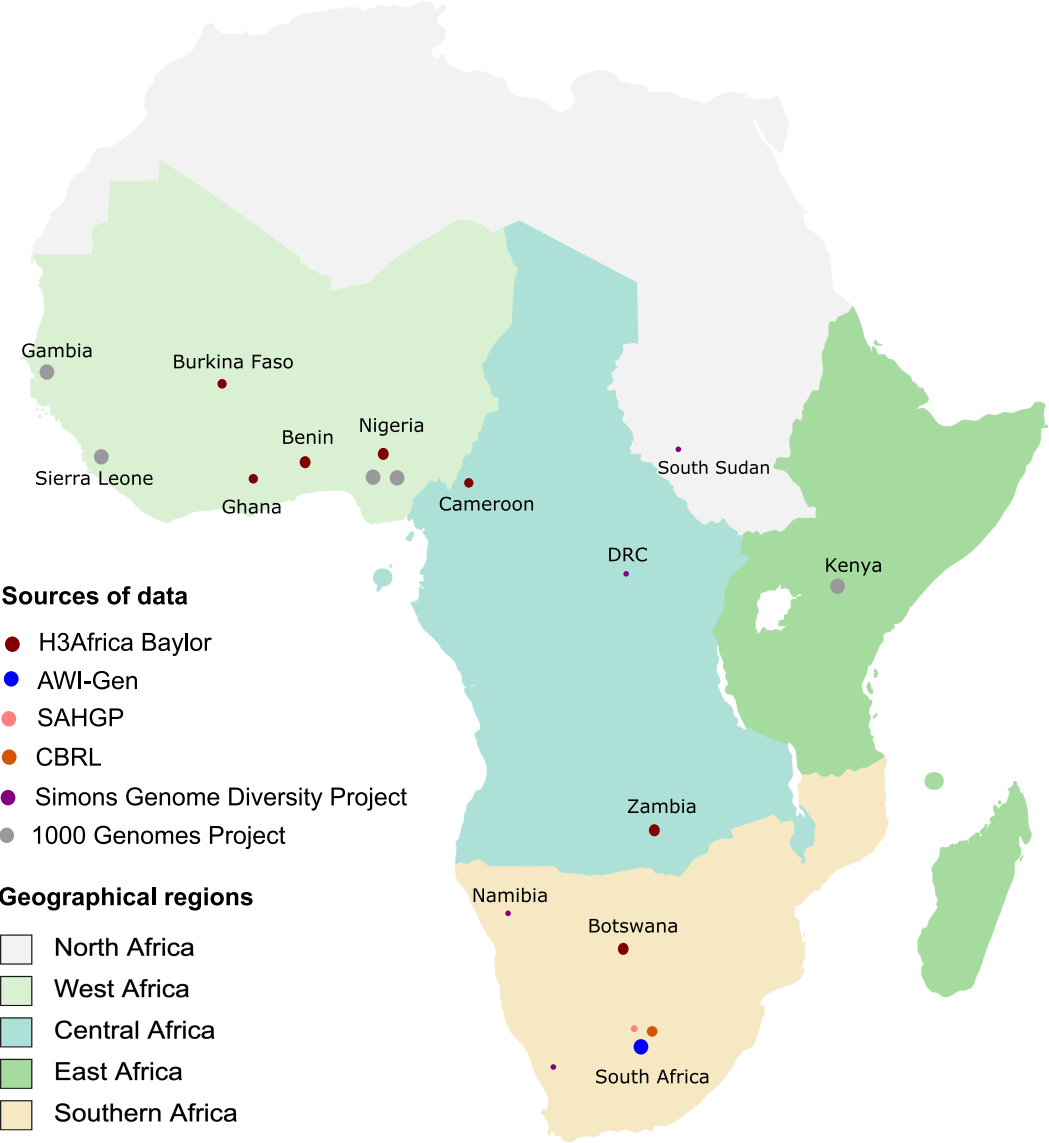
African geographical regions represented in the study. The size of the circles (not drawn to scale) reflect the relative number of participants from each population group. AWI‐Gen, Africa Wits‐INDEPTH partnership for Genomics studies; CBRL, Cell Biology Research Laboratory (National Institute of Communicable Diseases, Wits University, Johannesburg); DRC, Democratic Republic of the Congo; H3Africa, Human Heredity and Health in Africa Consortium; SAHGP, Southern African Human Genome Programme.

**Figure** **1** and **Table** **1** provide a summary of the African whole genome sequence data sets used in this study. These data were all sequenced at high‐depth (average depth of coverage = 30×) on Illumina platforms (San Diego, CA, USA). The bulk of the data sets are from the Human Heredity and Health in Africa (H3Africa) Consortium and the 1000 Genomes Project, mainly representing Niger‐Congo ethnolinguistic groups, with limited representation from Nilo‐Saharan and individuals of Khoe and San heritage.

**Table 1:** Sources of the high‐coverage whole genome sequence data sets used in the study

Populations	Project/Institution	*n*
H3Africa Consortium data		
Fon from Benin (FNB)	H3Africa Baylor; University of Montréal	50
Berom of Nigeria (BRN)	H3Africa Baylor; Institute of Human Virology	49
Cameroon (CAM)	H3Africa Baylor; University of Dschang	26
Ghana (GHA)	H3Africa Baylor; AWI‐Gen	26
Burkina Faso (BFA)	H3Africa Baylor; AWI‐Gen	33
South Africa	AWI‐Gen	100
Botswana (BOT)	H3Africa Baylor; CAfGEN	47
Bantu‐speakers from Zambia (BSZ)	H3Africa Baylor; University of Zambia	41
Data from other Africa‐based projects		
South Africa	SAHGP	15
South Africa	CBRL (NICD; Wits University)	40
African genomes in public repositories		
Botswana/Namibia	SGDP	3
Namibia	SGDP^a^	3
DRC	SGDP^b^	4
Gambia	SGDP	2
Kenya	SGDP^c^	5
Nigeria	SGDP	4
Senegal	SGDP	3
South Africa	SGDP^a^	3
South Sudan	SGDP^c^	3
Luhya in Webuye, Kenya (LWK)	1000 Genomes Project	99
Esan in Nigeria (ESN)	1000 Genomes Project	99
Yoruba in Ibadan (YRI)	1000 Genomes Project	108
Mende in Sierra Leone (MSL)	1000 Genomes Project	85
Gambian in Western Division, Mandinka (GWD)	1000 Genomes Project	113
Public data sets with genomes from other global superpopulations (for comparative analysis)		
African Caribbean in Barbados (ACB)	1000 Genomes Project	96
People with African Ancestry in Southwest United States (ASW)	1000 Genomes Project	61
European (EUR)	1000 Genomes Project	503
Admixed American (AMR)	1000 Genomes Project	347
South Asian (SAS)	1000 Genomes Project	489
East Asian (EAS)	1000 Genomes Project	504

The genomes of all the study participants were sequenced to an average read depth of ∼30× by the respective projects indicated in the table. AWI‐Gen, Africa Wits‐INDEPTH partnership for Genomics studies; CAfGen, The Collaborative African Genomics Network; CBRL, Cell Biology Research Laboratory; NICD, National Institute for Communicable Diseases; SAHGP, Southern African Human Genome Programme; SGDP, Simons Genome Diversity Project. The majority of the continental African participants referred to in this table are from the Niger‐Congo language family, except the following:

^a^ Khoe and San hunter‐gatherers.

^b^ Pygmy hunter‐gatherers.

^c^ Nilo‐Saharan (*n* = 2 for the Kenyan SGDP participants).

The H3Africa‐Baylor data set comprises WGS data from 272 individuals who were enrolled by various H3Africa studies. These individuals are representative of populations from Benin (FNB, Fon in Benin; *n* = 50), Botswana (BOT; *n* = 47), Cameroon (CAM; *n* = 26), Nigeria (BRN, the Berom of Nigeria; *n* = 49), Zambia (BSZ, Bantu speakers in Zambia; *n* = 41), Ghana (GHA; *n* = 26) and Burkina Faso (BFA; *n* = 33). The details about the sample preparation and sequencing (Illumina 150 base pair (bp) reads; > 30× depth of coverage) have been previously described.^20^, ^21^

The Wits‐INDEPTH partnership for Genomic studies (AWI‐Gen) contributed WGS samples from 100 southeastern Bantu speakers (SEB) from South Africa.^14^ These were all high‐coverage genomes.

The data set from the Cell Biology Research Laboratory (CBRL) consists of 40 samples from South African participants (39 African ancestry and one of mixed ancestry). Details relating to sample preparation and Illumina sequencing (depth of coverage > 30×) have been previously described by da Rocha *et al*.^21^

We included 15 participants from the Southern African Human Genome Programme (SAHGP).^28^ These participants are from two Bantu‐speaking ethnolinguistic groups: Sotho‐Tswana speakers (*n* = 8) and Xhosa speakers (*n* = 7). The WGS data consist of 101 bp paired‐end reads at a depth of > 30× and ~ 314 bp insert size.

Among the publicly available WGS data, we included 30 high‐coverage (average depth of 43×) African genomes generated by the Simon's Genome Diversity Project (SGDP)^29^ in the study. These samples were from individuals that are representative of various ethnolinguistic groups in Congo, Namibia, Kenya, Senegal, Nigeria, Gambia, Sudan, and South Africa. We also analyzed 504 high‐coverage (> 30×) genomes from continental African populations represented in the 1000 Genomes Project data set.^30^ We included the rest of the global 1000 Genomes Project data set in our analysis for comparison of the *CYP2D6* allele distribution in resident African populations vs. that in global populations. These data (*n* = 2000) are representative of African American/Afro‐Caribbean (*n* = 157), European (*n* = 503), East Asian (*n* = 504), South Asian (*n* = 489), and admixed American (*n* = 347) participants.

### DNA samples for *CYP2D6* star allele validation

Genomic DNA from 192 study participants was used during our long‐read‐based *CYP2D6* star allele validation under ethics amendment terms in protocol M200993. This included DNA samples from the CBRL participants, the SEB AWI‐Gen participants, and aliquots of samples provided by AWI‐Gen to H3Africa Baylor (GHA and BFA participants).

### Star allele analysis

Given the known challenges related to genotyping *CYP2D6*, we called *CYP2D6* star alleles from the African WGS samples using five separate tools, namely StellarPGx (v1.2.4),^23^ Cyrius (v1.1),^26^ Aldy (v3.1),^24^ Stargazer (v1.0.8)^25^ and Astrolabe (v0.8.7.0).^27^ Thereafter, we obtained a consensus *CYP2D6* diplotype call‐set from the output of these algorithms (**Supplementary Materials** **S2**). For each participant we determined the consensus diplotype by considering star alleles called by at least two of the tools. In a few cases, for example, samples for which no concordant diplotype was obtained between at least two of the algorithms and samples that seemed to have challenging structural rearrangements between *CYP2D6* and *CYP2D7*, we performed manual visual inspection of the read depth plots output by Stargazer and SAMtools depth,^31^ as well as manual variant inspections on the Integrative Genomics Viewer.^32^

### Metabolizer phenotype prediction

The *CYP2D6* consensus diplotype calls were translated to phenotypes by using the standardized activity score system recommended by CPIC.^8^ For each haplotype, activity values were assigned as follows: 0, 0.5, 1, and > 1 (dependent on gene copies) for no‐function, decreased‐function, normal‐function, and increased‐function haplotypes, respectively. However, the CYP2D6*10 allele was assigned an activity value of 0.25 as recommended.^8^ The activity score for each diplotype was obtained by adding up the activity values of component haplotypes. The participants were then classified as either poor metabolizers (AS = 0), intermediate metabolizers (0 < AS < 1.25), normal metabolizers (1.25 ≤ AS ≤ 2.25), or ultrarapid metabolizers (AS > 2.25).^8^

### *CYP2D6* long‐range PCR

*CYP2D6* 6.6 kb‐long amplicons (Fragment A) that contain the *CYP2D6* gene as well as upstream and downstream noncoding regions were generated following the long‐range (XL) polymerase chain reaction (PCR) protocol described by Gaedigk *et al*.^33^, ^34^ with some modifications. The XL‐PCR forward and reverse primers were tailed with universal sequences (5′‐GCAGTCGAACATGTAGCTGACTCAGGTCAC‐3′ and 5′‐TGGATCACTTGTGCAAGCATCACATCGTAG‐3′, respectively) on the 5′ end to enable sample barcoding of pooled amplicons via a second PCR and subsequent multiplexing. For samples where a gene duplication was computationally inferred from the short‐read WGS, the duplicated *CYP2D6* gene copy was amplified with FragD^33^ primers which produced 8.6 kb fragments. FragD primers are also used to amplify *CYP2D6‐2D7* hybrid alleles (~ 10.2 kb). For samples where CYP2D6*13 was predicted, FragH primers^33^ were used to amplify the ~ 5 kb‐long *CYP2D7‐2D6* hybrid amplicons. Each 20 μL reaction mix contained 60–120 ng of genomic DNA, 10 μL of 2X LongAmp Taq ReadyMix (New England Biolabs, South Africa), 1 μL of 100% dimethyl sulfoxide (DMSO) (Sigma‐Aldrich/Merck, South Africa), and 1 μL each of 10 μM forward and reverse primers (Inqaba Biotech, South Africa). The details about the specific primers used to amplify the aforementioned XL‐PCR fragments and the PCR cycling conditions are provided in **Supplementary Materials** **S2**.

### Amplicon pooling and barcoding

Quality control of the PCR products from the first‐round PCR was carried out using a 0.8% agarose gel for visual inspections and the Agilent 4200 TapeStation (Agilent, Santa Clara, CA, USA) for quantification using the D5000 Screen tape kit. For each participant, *CYP2D6* amplicons were pooled equimolarly with *CYP2A6* and *CYP2B6* amplicons from a related subproject. All amplicons were in the size range of 5–12 kb. The amplicon pools were purified using the AMPure PB bead purification (Pacific Biosciences, Menlo Park, CA, USA), Thereafter, barcodes were added to the purified amplicons via a second round of PCR. The 25 μL reaction mix contained 5–10 ng/μL of initial pooled PCR product, 11 μL of 2X longAmp Taq ReadyMix (New England Biolabs, Ipswich, MA, USA), 1 μL of DMSO (100%), and 5.0 μL of 2 μM barcoded universal primers (Inqaba Biotech, Pretoria, South Africa). The PCR cycling conditions were as follows: 20 cycles of 95°C for 1 minute, 65°C for 30 seconds, 72°C for 11 minutes.

### Single‐Molecule Real‐Time sequencing

As with amplicon pooling and barcoding, high‐fidelity (HiFi) sequencing (Pacific Biosciences) for the *CYP2D6* XL‐PCR amplicons was outsourced to Inqaba Biotech (Pretoria, South Africa). SMRTbell libraries were constructed from ~ 500 ng of pooled fragments by following standard end‐repair, adapter ligation, and purification strategies detailed in the Pacific Biosciences protocols (https://www.pacb.com/wp‐content/uploads/Procedure‐Checklist‐Preparing‐HiFi‐SMRTbell‐Libraries‐using‐SMRTbell‐Express‐Template‐Prep‐Kit‐2.0.pdf). Annealing and binding of SMRTbell templates was performed using the Sequel II Binding kit 2.2 and sequencing primer v5, and circular consensus sequencing was performed for a movie time of 30 hours to generate HiFi reads via the SMRT Link software on the Sequel IIe instrument (Pacific Biosciences) at Inqaba Biotech (Pretoria, South Africa).

Raw HiFi sequence data were demultiplexed and processed into individual samples according to the corresponding barcode sequences using the NGSutils next‐generation sequencing data analysis software kit.^35^ *CYP2D6* HiFi reads were aligned to the *CYP2D6* region in GRCh38 and GRCh37, and also to the NG_008376.4 (LRG_303) reference sequence using pbmm2 v1.7.0 (https://github.com/PacificBiosciences/pbmm2). Variant calling was done using DeepVariant.^36^ Thereafter, variant phasing and read haplotagging was carried out for samples containing more than one heterozygous variant using WhatsHap.^37^

### Variant functional prediction

*CYP2D6* variants were annotated using the Ensembl Variant Effect Predictor (VEP).^38^ The functional effects (corresponding to the NM_000106.6 transcript) of potential novel star allele‐defining variants were predicted using VEP plugins including sorting intolerant from tolerant,^39^ Polyphen‐2,^40^ combined annotation‐dependent depletion,^41^ likelihood ratio test,^42^ protein variation effect analyzer,^43^ and variant effect scoring tool v4,^44^ taking into account the ADME‐optimized parameters suggested by Zhou *et al*.^45^ sorting intolerant from tolerant Indel^46^ was used to annotate frameshift variants while loss‐of‐function transcript effect estimator^47^ was used to identify loss of function variation.

### Statistical analysis

Allele frequencies were calculated and tested for deviation from Hardy–Weinberg equilibrium using the Fisher exact test in R (R Foundation for Statistical Computing) (https://www.r‐project.org). The Fisher exact test was also used to determine significant differences in population *CYP2D6* allele and diplotype frequencies. *P* values of < 0.05 were considered statistically significant. Allele frequency data for previous studies across SSA was obtained from the Pharmacogenomics Knowledgebase (PharmGKB) (https://www.pharmgkb.org/page/cyp2d6RefMaterials).

## RESULTS

### Sample populations

Our study data set was mainly representative of Niger‐Congo ethnolinguistic groups (**Table** **1**) (see also previous analyses by Choudhury *et al*. and da Rocha *et al*.). Only a few genomes (*n* = 5) from Nilo‐Saharan populations were analyzed in this study. There is a modest representation of participants (*n* = 6) with Khoe and San heritage. The genomes of the Khoe and San are typically deeply divergent from those of Niger‐Congo speakers.^22^ Results from the *CYP2D6* star allele analysis of African American/Afro Caribbean (ASW and ACB), European (EUR), admixed American (AMR), South Asian (SAS), and East Asian (EAS) populations represented in the 1000 Genomes Project high coverage data set^30^, ^48^ are included for comparison.

### *CYP2D6* star allele frequencies

For previously characterized alleles, the nomenclature used here is in accordance with PharmVar definitions (https://www.pharmvar.org/gene/CYP2D6). All *CYP2D6* variant denotation follows the numbering based on NG_008376.4 counting from the sequence start, and/or protein change where applicable.

Of the known *CYP2D6* star alleles cataloged by PharmVar (https://www.pharmvar.org), 38 distinct star alleles were detected in the SSA data sets in this study, including 11 *CYP2D6* structural variants (**Tables** **2** and **3**). These *CYP2D6* SVs (*1xN, *2xN, *4xN, *5, *13, *17x2, *29x2, *36, *43x2, *45xN, *68 + *4) accounted for 14% of the *CYP2D6* haplotypes called from SSA populations (see **Supplementary Materials** **S3**). Notably, some star alleles considered under the SNV‐defined allele bracket are known to harbor smaller benign SVs e.g., the *CYP2D6* intron 1 conversion to *CYP2D7* (https://www.pharmvar.org).

**Table 2:** *CYP2D6* star allele frequencies

		Allele frequencies (%)						
*CYP2D6* allele	CPIC function	SSA in this study (*n* = 947)	Previous SSA studies^a^ (*n* = 2,248)	African American/Afro Caribbean (*n* = 157)	EUR (*n* = 498)	AMR (*n* = 345)	SAS (*n* = 481)	EAS (*n* = 502)
*1	Normal	24.7	7.8	31.5	35.8	45.1	39.5	26
*2	Normal	13.4	19.8	9.6	15.9	18.3	20.8	7.8
*27	Normal	0.3	0.5	0.3	0	0.3	0	0
*34	Normal	0.1		0	0	0	0	0
*39	Normal	0.1	0	0.3	0.1	0.1	0.4	0.3
*45	Normal	3.7	4.2	2.2	0	0.3	0	0
*46	Normal	0.5	0.2	1	0	0.1	0	0
*17x2	Normal	0.2		0	0	0	0	0
*29x2	Normal	0.3		0.3	0	0	0	0
*41x2	Normal	0	0.2	0	0	0	0	0
*10	Decreased	3.9	5.6	3.8	1.2	1.6	3.5	15.2
*17	Decreased	19.5	19.3	16.9	0.2	0.9	0	0
*29	Decreased	10	12.1	5.7	0	0.3	0	0
*41	Decreased	0.8	11.5	2.9	8.5	6.1	11.4	3.4
*84	Decreased	0.1		0.3	0	0	0	0
*3	No function	0	0.2	1	1.8	0.6	0.2	0
*4	No function	1.7	3.4	4.1	11.8	9.6	8.2	0.2
*4x2	No function	2.2		3.8	0.3	0.1	0	0
*4x3	No function	0.1		0.1	0	0	0	0
*5	No function	8.1	5.2	7	2.4	2.2	2.5	3.5
*12	No function	0.2	0.3	0	0	0	0	0
*13	No function	0.1		0.3	0.2	0.1	0.1	0
*15	No function	0.2	0.6	0	0	0	0	0
*36	No function	0.4		0.3	0	0	0	0.2
*40	No function	1.4	1.3	0.3	0	0	0	0
*42	No function	0.1		0	0	0	0	0
*56	No function	0.2		0	0	0	0	0
*69	No function	0.1		0	0.1	0	0.2	0.3
*68 + *4	No function	0.1		1	5.7	2.6	2.2	0
*1x2	Increased	0.4	1.1	0.3	0.5	1.2	0.6	0.3
*1x3	Increased	0.2		0	0	0.1	0	0
*2x2	Increased	1.9	1.7	1.3	1.5	0.6	0.4	0.5
*2x3	Increased	0.1		0	0.1	0	0	0.1
*2x4	Increased	0.2		0	0	0	0	0
*45x2	Increased	0.1		0	0	0	0	0
*45x3	Increased	0.1		0	0	0	0	0
*32	Uncertain	0	0.3	0	0.3	0	0.2	0
*43	Uncertain	0.8	1.7	1	0.1	0	1	0
*43x2	Uncertain	0.1		0	0	0.1	0	0
*52	Uncertain	0	0.8	0	0	0	0	0.1
*73	Unknown	0.2	0.5	0	0	0	0	0
*74	Unknown	0.1	0.5	0	0	0	0	0
*106	Unknown	1.5		0	0	0.1	0	0
*122	Unknown	0.1		0.3	0	0	0	0
*125	Unknown	0.3		0.3	0	0	0	0
*139	Unknown	0.1		0	0	0	0.1	0
*149	Unknown	0.3		0	0	0	0	0.1

*CYP2D6* star allele frequencies in sub‐Saharan African populations compared with previous studies in this region and global frequency distributions. See **Supplementary Materials** **S3** for the full list including *CYP2D6* star alleles called in the global biogeographical groups but not in SSA.

AMR, Admixed American; CPIC, Clinical Pharmacogenetics Implementation Consortium; EAS, East Asian; EUR, European; n, individuals; PharmGKB, Pharmacogenomics Knowledge Base; SSA; sub‐Saharan Africa; SAS, South Asian.

^a^ See PharmGKB and CPIC *CYP2D6* reference materials (https://www.pharmgkb.org/page/cyp2d6RefMaterials).

**Table 3:** *CYP2D6* star allele frequencies across all sub‐Saharan African populations included in the study

		Allele frequencies (%)													
*CYP2D6* allele	CPIC function	All (*n* = 947)	FNB (*n* = 49)	BRN (*n* = 49)	BFA (*n* = 33)	GHA (*n* = 26)	CAM (*n* = 26)	BSZ (*n* = 41)	BOT (*n* = 47)	SA (*n* = 153)	ESN (*n* = 98)	YRI (*n* = 107)	GWD (*n* = 111)	MSL (*n* = 84)	LWK (*n* = 95)
*1	Normal	24.7	17.3	24.5	30.3	26.9	21.2	32.9	31.9	22.9	29.6	25.7	26.6	18.5	20.5
*2	Normal	13.4	9.2	15.3	13.6	13.5	21.2	12.2	11.7	13.7	9.2	11.2	14	14.3	18.4
*27	Normal	0.3	1	0	0	0	0	0	0	0	0	0	0	0	2.1
*34	Normal	0.1	0	0	0	0	0	0	0	0	0	0	0.5	0	0
*39	Normal	0.1	1	0	0	0	0	0	0	0	0	0	0	0	0
*45	Normal	3.7	4.1	3.1	4.5	7.7	3.8	3.7	2.1	4.9	4.6	3.3	1.8	3.6	3.7
*46	Normal	0.5	0	1	0	0	0	0	1.1	0	1	0	1.4	0	1.1
*17x2	Normal	0.2	1	0	1.5	0	0	0	0	0	0.5	0	0	0	0
*29x2	Normal	0.3	2	0	0	0	0	0	0	0	0	0	1.4	0	0.5
*10	Decreased	3.9	5.1	3.1	1.5	0	1.9	4.9	3.2	4.6	1.5	4.7	5.4	8.9	1.1
*17	Decreased	19.5	30.6	23.5	15.2	21.2	5.8	14.6	19.1	16.7	22.4	22.9	14.9	23.8	18.4
*29	Decreased	10.0	4.1	10.2	12.1	5.8	13.5	17.1	6.4	10.1	7.1	9.3	9.9	8.9	16.3
*41	Decreased	0.8	2	0	0	0	0	0	0	0.7	1.5	0.9	0.5	0.6	2.6
*84	Decreased	0.1	0	1	0	0	0	0	1.1	0	0	0	0	0	0
*4	No function	1.7	3.1	0	0	0	3.8	1.2	0	2.3	2.6	0.5	2.7	1.2	2.1
*5	No function	8.1	7.1	6.1	6.1	7.7	7.7	7.3	13.8	14.7	3.6	6.5	5	8.9	4.7
*12	No function	0.2	0	0	0	0	1.9	0	2.1	0	0	0	0	0	0
*15	No function	0.2	0	0	0	0	0	0	0	0	1	0.5	0	0	0
*36	No function	0.4	0	0	1.5	0	0	0	0	0	0	0	1.8	1.2	0
*40	No function	1.4	1	1	0	1.9	1.9	0	2.1	2.6	2.6	0.5	0.9	1.8	0
*42	No function	0.1	0	0	0	0	0	0	0	0	0	0	0.5	0	0.5
*56	No function	0.2	0	0	0	0	0	2.4	0	0	0	0	0	0.6	0
*69	No function	0.1	0	0	0	0	0	0	0	0.3	0	0	0	0	0
*68 + *4	No function	0.1	0	0	0	1.9	0	0	0	0	0	0	0	0	0
*13	No function	0.1	0	0	1.5	0	0	0	0	0	0	0	0	0	0
*4x2	No function	2.2	2	2	3	3.8	3.8	1.2	0	1.3	5.1	4.7	1.4	1.8	0.5
*4x3	No function	0.1	0	0	0	0	0	0	0	0	0	0.5	0	0.6	0
*1x2	Increased	0.4	0	1	0	3.8	0	0	1.1	0	0	0.5	1.4	0	0
*1x3	Increased	0.2	0	0	0	0	0	0	0	0.7	0	0.5	0	0	0
*2x2	Increased	1.9	2	3.1	3	0	1.9	0	3.2	0	5.1	2.8	2.3	1.2	1.1
*2x3	Increased	0.1	0	0	0	0	0	0	0	0	0	0	0	0.6	0
*2x4	Increased	0.1	2	1	0	0	0	0	0	0	0	0	0	0	0
*45x2	Increased	0.1	0	0	0	0	1.9	0	0	0	0	0	0	0	0.5
*45x3	Increased	0.1	0	0	0	0	0	0	0	0	0	0	0	0	0.5
*43	Uncertain	0.8	0	2	1.5	1.9	0	0	0	0.3	0.5	0	1.8	0	3.2
*43x2	Uncertain	0.1	0	0	0	0	0	0	0	0	0	0.5	0	0	0
*73	Unknown	0.2	0	0	0	0	0	0	0	1	0	0	0	0	0
*74	Unknown	0.1	0	0	0	0	0	0	0	0.3	0	0	0	0	0
*106	Unknown	1.5	5.1	0	3	1.9	3.8	1.2	0	0.7	1	3.3	1.8	1.2	0
*122	Unknown	0.1	0	0	0	0	0	0	0	0	0	0	0.5	0	0
*125	Unknown	0.3	0	1	0	0	0	0	0	0	0.5	0.5	0.5	0	0.5
*139	Unknown	0.1	0	0	0	0	0	0	0	0	0.5	0	0	0	0
*149	Unknown	0.3	0	0	1.5	1.9	0	0	0	0	0	0	0.9	0.6	0

BFA, participants from Burkina Faso; BOT, participants from Botswana; BRN, Berom in Nigeria; BSZ, Bantu‐speakers in Zambia; CAM, Cameroonian participants; ESN, Esan in Nigeria; FNB, Fon in Benin; GHA, Ghanaian participants; GWD, Gambian in Western Division (Mandinka); LWK, Luhya in Webuye (Kenya); MSL, Mende in Sierra Leone; SA, South African participants; YRI, Yoruba in Ibadan, Nigeria.

Of the continental African study participants, 27% harbored at least one SV‐defined *CYP2D6* star allele. This was similar to the proportion of individuals with *CYP2D6* SVs in the ASW (29.5%) and ACB (28%) populations, higher than the proportion in European participants (22.7%), significantly higher than the proportion in admixed American participants (17%, *P* = 0.00015) and South Asian participants (14.1%, *P* = 1.4 × 10^−8^), and significantly less than the proportion in East Asian participants (66.3%, *P* < 2.2 × 10^−16^).

Among the known normal‐function star alleles, CYP2D6*1 (reference allele, but known to have many suballeles) was the most frequent in SSA, followed by CYP2D6*2 and CYP2D6*45 (**Table** **2**). The CYP2D6*1 frequency observed in SSA in this study (24.7%) is higher than the average frequency (7.8%) from previous SSA studies curated by PharmGKB, while the reverse is true for the observed SSA CYP2D6*2 frequency (13.4%) in this study. CYP2D6*45 is largely African‐specific as expected (**Table** **2**). The CYP2D6*17x2 and CYP2D6*29x2 structural variant alleles found in SSA are grouped under normal‐function alleles as the two copies combined make up for the function deficiency of individual CYP2D6*17 and *29 alleles, respectively.

We detected CYP2D6*17, *29, *41, and *84 in SSA among the known decreased function *CYP2D6* star alleles. As expected, CYP2D6*17, which is defined mainly by 6041C>T (T107I), was the most frequent decreased function allele found in SSA participants and among the African American / Afro‐Caribbean individuals, followed by CYP2D6*29 (**Table** **2**). CYP2D6*17 was found to be most frequent among the Fon from Benin (AF = 30%) and significantly less frequent among the participants from Cameroon (AF = 5.8%) compared with participants from both neighboring and distant groups within SSA (**Table** **3**). CYP2D6*29, which is defined mainly by 6679G>A+6681G>C (V136I) and 8203G>A (V338M), was also nonuniformly distributed across SSA (**Table** **3**). In addition, the frequency of CYP2D6*29 was found to be significantly lower (*P* = 0.01598) in African American / Afro‐Caribbean participants (combined AF = 5.7%) compared with the average frequency in SSA (**Table** **2**). Notably, the observed frequency of CYP2D6*41 in our SSA cohorts (AF = 0.8%) was significantly less than the average frequency (11.5%) calculated by the PharmGKB from previous studies in the region (see https://www.pharmgkb.org/page/cyp2d6RefMaterials). Also, none of the SSA participants with the CYP2D6*10 allele had the *36 allele in tandem.

Among the no‐function *CYP2D6* alleles, we found the *CYP2D6* full gene deletion (CYP2D6*5) to be more frequent in SSA (AF = 8.1%) compared with previously reported frequencies^5^ and to all the other global biogeographical groups (**Table** **2**). CYP2D6*5 accounted for over 52% of the no‐function haplotypes called from SSA populations, and it occurred most frequently among the participants from Botswana (AF = 13.8%) and South Africa (AF = 14.7%), but it was less frequent among the East and West African population groups, e.g., LWK (AF = 4.7%) and ESN (AF = 3.5%), respectively (**Table** **3**). In addition, the CYP2D6*5/*5 diplotype (i.e., homozygous *CYP2D6* gene deletion) was observed in 10 SSA individuals, including 3 from Botswana and 3 from South Africa, but it was virtually absent across other global biogeographical groups. The CYP2D6*4 allele defined by rs3892097 (splice defect) occurred at a significantly lower frequency (1.7%) in the SSA populations in this study compared with the frequency in European populations (AF = 11.8%) and was also less frequent than the estimated average frequency from previous studies in SSA (*P* = 0.0002; **Table** **2**). Conversely, we found CYP2D6*4xN alleles to be more frequent in SSA than in European populations, which have a higher frequency of CYP2D6*68+*4 and the rarer CYP2D6*4+*4.013. The virtually African‐specific no‐function star alleles called in SSA diplotypes were CYP2D6*12, *15, *40, *42, and *56. The no‐function hybrid alleles which were detected, albeit rarely in SSA, include CYP2D6*13, CYP2D6*36, and CYP2D6*68+*4 (**Tables** **2** and **3**).

The increased‐function *CYP2D6* star alleles identified in SSA in this study include CYP2D6*1xN, *2xN, and *45xN. These accounted for 20% of the *CYP2D6* structural variants identified in SSA and were generally more frequent in western African populations compared with southern African populations (**Table** **3**). For example, CYP2D6*2x2 was absent from Zambian and South African participants but had a frequency of ≥ 2% among the FNB, BFA, CAM, ESN, YRI, and GWD populations. However, this allele was found to be frequent in Botswana (AF = 3.2%) as well.

**Figure 2:**
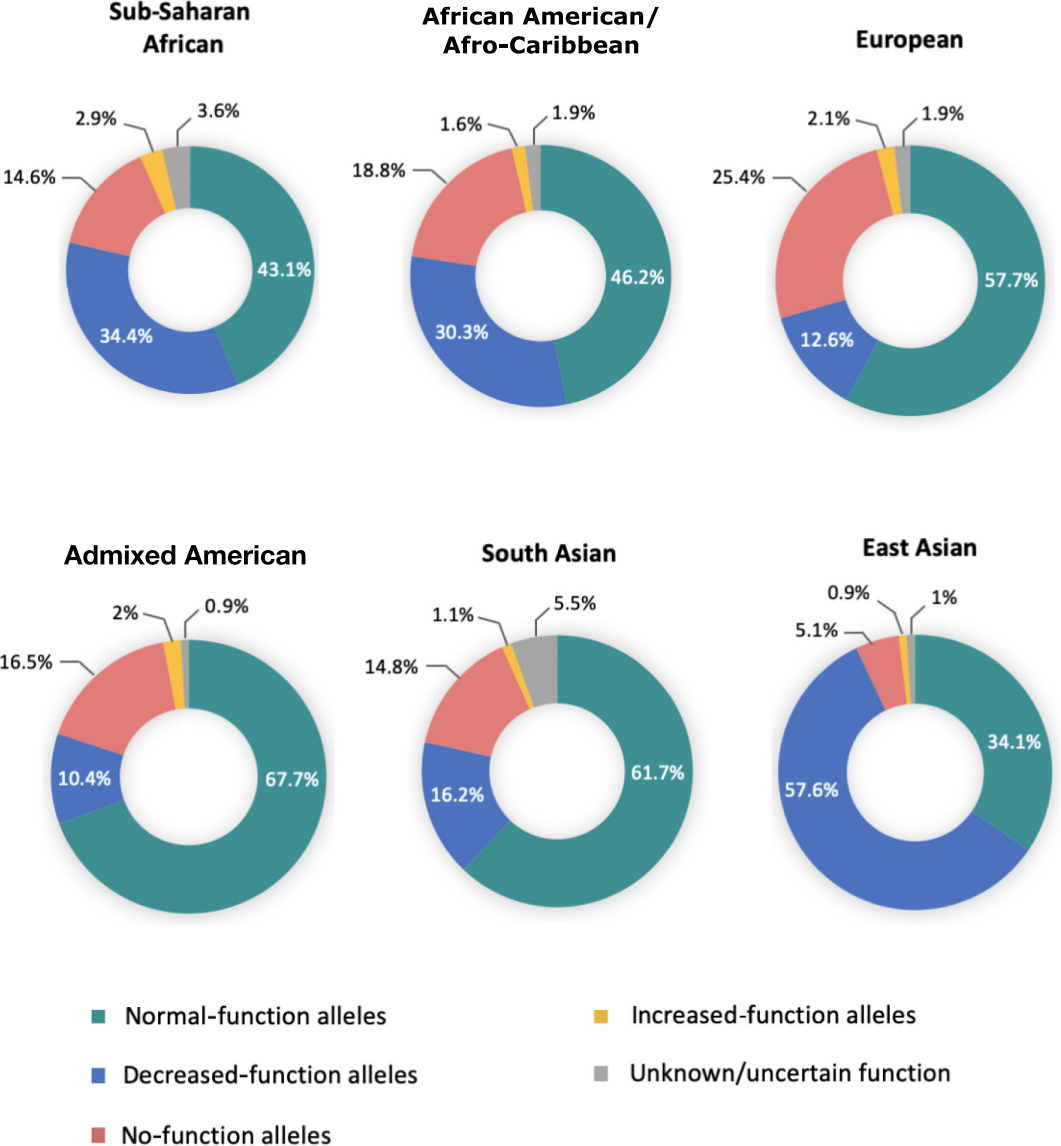
Frequency distribution of *CYP2D6* star alleles grouped by function across African and global populations. SSA populations had a higher frequency of decreased‐function *CYP2D6* star alleles compared with European, admixed American, and South Asian populations in part due to a higher frequency of CYP2D6*17 and *29. Conversely, CYP2D6*10 accounts for the high proportion of decreased‐function alleles in East Asian populations. SSA and South Asian populations had a relatively higher proportion of alleles with unknown or uncertain function which warrants more functional assays to determine the CPIC function of these alleles. The sample sizes were as follows: sub‐Saharan African = 961 participants; African American/Afro‐Caribbean = 157 participants; European = 503 participants; Admixed American = 347 participants; South Asian = 489 participants; East Asian = 504 participants. CPIC, Clinical Pharmacogenetics Implementation Consortium.

Regarding the *CYP2D6* star alleles with unknown/uncertain function, these were more frequent in SSA (AF = 3.6%) and South Asian (AF = 5.5%) populations compared with other global biogeographical groups (**Figure** **2**). Across SSA, the star alleles in this category were the relatively common CYP2D6*106 (SSA AF = 1.5%), and *73, *74, *125, and *149, which were relatively rare (**Table** **2**). The CYP2D6*43 allele was most frequent among the LWK (AF = 3.2%) compared with other SSA populations (**Table** **3**).

Overall, SSA populations were found to have a statistically significant higher frequency of decreased function *CYP2D6* star alleles compared with European (*P* = 2.2 × 10^−16^), admixed American (*P* = 2.2 × 10^−16^), and South Asian (*P* = 2.2 × 10^−16^) populations (**Figure** **2**) largely due to the high prevalence of the African‐ancestry‐associated CYP2D6*17 and *29 alleles. However, East Asian populations had the highest frequency of decreased‐function *CYP2D6* star alleles (57.6%), mainly due to the high occurrence of CYP2D6*10, which is often involved in various tandem rearrangements with the *36 hybrid (**Supplementary Materials** **S3**).

### CYP2D6 phenotype distribution

**Figure 3:**
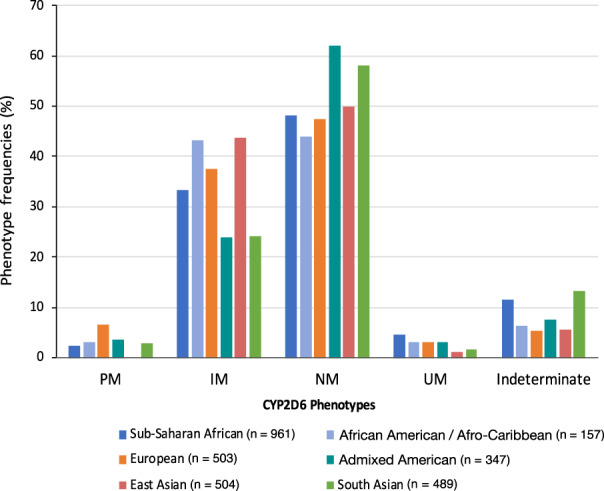
Distribution of CYP2D6 phenotypes predicted from diplotypes called from high‐coverage WGS data. IM, intermediate metabolizer; n, sample size; NM, normal metabolizer; PM, poor metabolizer; UM, ultrarapid metabolizer; WGS, whole genome sequence.

**Figure 4:**
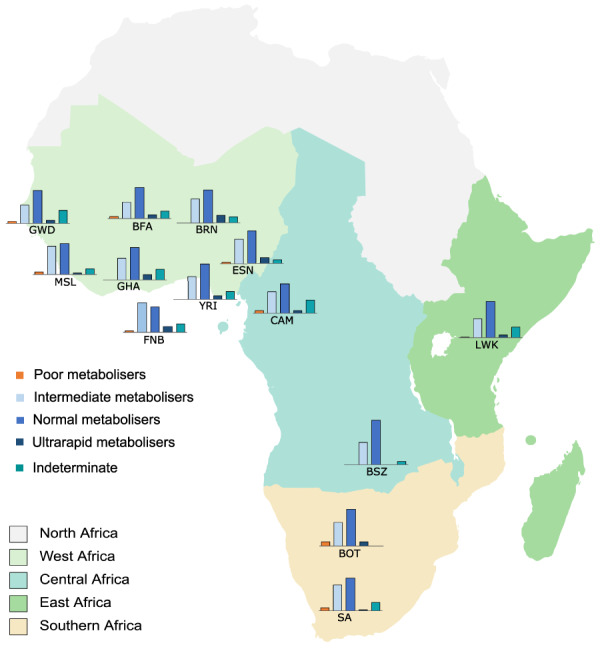
CYP2D6 phenotype distribution across the SSA populations in the study. BFA, participants from Burkina Faso; BOT, participants from Botswana; BRN, Berom in Nigeria; BSZ, Bantu‐speakers in Zambia; CAM, Cameroonian participants; ESN, Esan in Nigeria; FNB, Fon in Benin; GHA, Ghanaian participants; GWD, Gambian (Mandinka) in Western Divisions  of the Gambia; LWK, Luhya in Webuye (Kenya); MSL, Mende in Sierra Leone; SA, South African participants (south eastern Bantu‐speakers); SSA, sub‐Saharan African; YRI, Yoruba in Ibadan, Nigeria.

The predicted CYP2D6 metabolizer phenotype distributions, translated from the *CYP2D6* diplotype calls using the AS system, are highlighted in **Figures** **3** and **4**. Twenty‐two participants (2.3%) were predicted to be CYP2D6 poor metabolizers (PMs) across the SSA populations in the study (**Figure** **3**). There were no significant differences between the PM phenotype distributions across SSA. Each SSA population in the study had at least one CYP2D6 PM except for the BRN, YRI, GHA, and BSZ (**Figure** **4**). The highest frequencies of PM diplotypes responsible for the PM phenotype were observed among participants from Botswana (6.4%) and South Africa (3.9%). In comparison, the CYP2D6 PM phenotype was common among the Europeans (6.6%) mainly due to CYP2D6*4, and no occurrence was observed among the East Asian participants (**Figure** **3**).

For the intermediate metabolizer (IM) phenotype, considerable differences in proportions were observed across SSA ranging from 26.5% in the GWD to 42% in the FNB (**Figure** **4**). This includes a statistically significant difference between the MSL and GWD (*P* = 0.033). The average frequency of diplotypes predicting the IM phenotype across SSA (33.9%) was found to be less than that in African American / Afro‐Caribbean (43.3%, *P* = 0.0244), European (37.6%, *P* = 0.1671, i.e., not statistically significant), and East Asian (43.7%, *P* = 0.00027) participants, but significantly higher than the frequency in admixed American (23.9%, *P* = 0.0006) and South Asian participants (24.1%, *P* = 1.4 × 10^−4^; **Figure** **3**).

We observed a higher frequency of diplotypes predicting the ultrarapid metabolizer phenotype in SSA (4.7%) compared with that in other global biogeographical groups based on the data sets analyzed in this study (**Figure** **3**). These diplotypes were disproportionately more frequent among participants from western Africa compared with east, central, and southern Africa (**Figure** **4**), with the highest frequencies being among the Berom (10.2%) and the Esan (9.1%) in Nigeria, and the lowest frequencies being among the BSZ (0%) and the South African participants (1.3%). These frequency differences (highest vs. lowest) were statistically significant.

### Potential novel *CYP2D6* star alleles inferred from short‐read WGS data

We identified 27 potential novel *CYP2D6* star alleles in 5% of the SSA participants. Nineteen of these predicted novel star alleles were fully phased computationally (**Table** **4**) while 8 were observed in individuals whose diplotypes could not be resolved (**Supplementary Materials** **S3**). Although all the core variants for these novel star alleles were found to be already cataloged in the Single‐Nucleotide Polymorphism Database (dbSNP), the haplotypes themselves had not been assigned star allele (or suballele) status by PharmVar at the time of our analysis and were thus considered as novel. For the phased potential novel alleles, the phasing was made possible either by having the same background allele on both chromosomes or observing multiple participants with the same novel core variant combination. However, suballeles could not be determined using this approach, so the potential novel alleles are indicated in **Table** **4** using only the core variants. These core variants were all found to be deleterious by at least one of the VEP plugins used in the study, except for rs140900383 (A226V), rs141756339 (R474Q), and rs374616348 (V119L) (**Supplementary Materials** **S3**). Potential novel *CYP2D6* star alleles inferred from global biogeographical groups other than SSA are highlighted in **Supplementary Materials** **S3**.

**Table 4:** Potential novel African ancestry *CYP2D6* star alleles inferred from short‐read high‐coverage whole genome sequence data

Haplotype	Background allele	Additional core variant(s)	Variant type	Count	Country / data set^a^
1	*1	rs140900383∼7471C>T	Missense (A226V)	1	Gambia (1000G)
2	*1	rs141756339∼9164G>A	Missense (R474Q)	1	Nigeria
3	*1	rs565013903∼7600G>A	Missense (R269P)	3	Namibia (SGDP)
4	*1	rs567606867∼7004G>A	Missense (E215K)	1	Sierra Leone (1000G)
5	*2	rs368858603∼7610_7611insT	Frameshift (T272TX)	2	Botswana; Kenya (1000G)
6	*2	rs368858603∼7610_7611insT + rs374616348∼6628G>T	Frameshift/stop‐gained (T272TX) + Missense (V119L)	2	Cameroon; Congo (SGDP)
7	*2	rs28371704∼6002A>G + rs28371703∼5992C>A	Missense (H94R, L91M)	1	Kenya (1000G)
8	*2	rs375715419∼9115A>G	Missense (T458A)	1	Zambia
9	*2	rs376636053∼6655T>C	Missense (W128R)	1	Sierra Leone (1000G)
10	*2	rs769157652∼8873G>A	Missense (E410K)	1	Sierra Leone (1000G)
11	*17	rs747089665∼8885C>T + rs769157652∼8873G>A	Missense (R414C, E410K)	1	South Africa
12	*17	rs1450231864∼8231T>C	Missense (M347T)	1	South Africa (SGDP)
13	*29	rs76802407~6012C>G^b^	Missense (D97E)	5	Burkina Faso, Ghana; Gambia, Sierra Leone (1000G)
14	*29	rs201006451∼6767C>T	Missense (A165V)	1	Nigeria (1000G)
15	*29	rs536109057∼5173C>T	Stop‐gained	2	Gambia (1000G)
16	*29	rs760940331∼9096G>A	Missense (M451I)	2	Gambia (1000G), Nigeria (1000G)
17	*41	rs141824015∼8206A>C	Missense (I339L)	4	Kenya (1000G), South Africa
18	*45	rs3915951∼8177G>T	Missense (R329L)	2	Gambia (1000G)
19	*70	rs16947∼7870C>T^c^	Missense (R296C)	5	Cameroon, South Africa

SGDP, Simons Genome Diversity Project; 1000G, 1000 Genomes Project.

^a^ IDs for Coriell and SGDP samples are provided in **Supplementary Materials** **S3** as these samples can potentially be used as reference materials.

^b^ This haplotype has recently been validated by Gaedigk *et al*.^7^—see published haplotype at https://www.pharmvar.org/gene/CYP2D6. Confirmatory submissions to PharmVar for the suballeles identified in this study are ongoing.

^c^ Matimba *et al*.^16^ identified CYP2D6*70 (moderate evidence on PharmVar) in their study. In our analysis and laboratory validation, we found that individuals with the *70‐defining variants also had 7870C>T (R296C) in *cis*.

### *CYP2D6* novel allele characterization using targeted long‐read sequencing

*CYP2D6* XL‐PCR was used to generate fragments for long‐read sequencing involving 190 participants, 32 from Burkina Faso, 26 from Ghana, and 132 from South African studies (AWI‐Gen and CBRL). For one participant who was predicted to have CYP2D6*13/*45, the *13 CYP2D7‐2D6 hybrid fragment did not amplify—we could not resolve whether the issue related to sample quality or absence of CYP2D6*13. For all the other participants, the corresponding XL‐PCR *CYP2D6* fragments were successfully amplified in preparation for subsequent long‐read sequencing.

From the three batches of samples (*n* = 189) sent for *CYP2D6* HiFi sequencing (along with *CYP2A6* and *CYP2B6* XL‐PCR fragments from a related study), *CYP2D6* XL‐PCR fragments were successfully barcoded for only 141 of these participants. Generally, the *CYP2D6* diplotypes derived from targeted PacBio HiFi resequencing were concordant to those inferred from short‐read‐based calls across all the samples included in the laboratory validation study.

**Figure 5:**
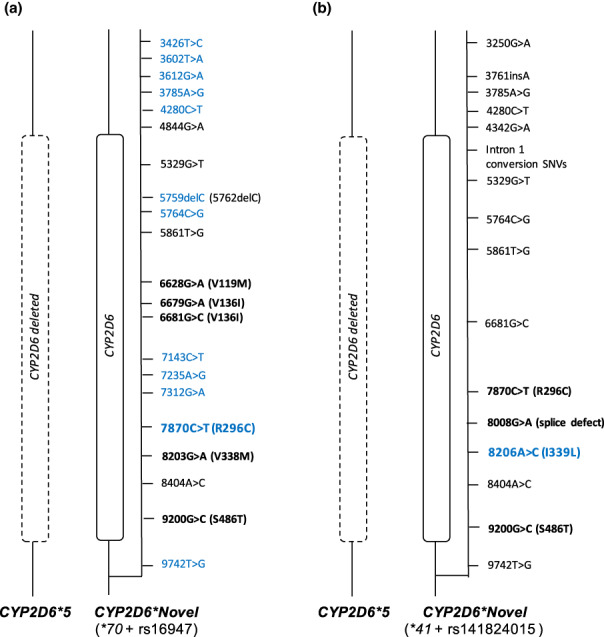
Novel *CYP2D6* star alleles in SSA characterized via XL‐PCR and HiFi sequencing. Panel (**a**) shows the two haplotypes in a South African south eastern Bantu‐speaking participant with the novel CYP2D6*70+rs16947 (R296C) haplotype. The submission of this allele for consideration by PharmVar is ongoing. The variant phasing and frequency information from our analysis suggests that CYP2D6*70, which has a “moderate” PharmVar level of evidence, should be redefined to include rs16947 but the final decision on this rests with the PharmVar *CYP2D6* expert panel. Panel (**b**) shows the two alleles identified in a South African participant with the novel CYP2D6*41+rs141824015 (I339L) allele. Two suballeles of this haplotype have been predicted in the Luhya in Webuye, Kenya (LWK) from the 1000 Genomes Project data set and they are being characterized in a collaborative project with 3 labs led by PharmVar members. SNVs (single‐nucleotide variations) indicated in black text are from the “backbone” haplotype. HiFi, high fidelity; PharmVar, Pharmacogene Variation Consortium; SSA, sub‐Saharan Africa; XL‐PCR, long‐range polymerase chain reaction.

HiFi sequencing enabled the characterization of two predicted novel major alleles (**Figure** **5**) in this batch of samples and revealed novel suballeles in multiple participants (see **Supplementary Materials** **S3** for examples).

**Case 1:** Referring to the short‐read‐based allele calling, StellarPGx and the other algorithms used could not assign a diplotype to three of the individuals carrying CYP2D6*70 core SNVs, while two other participants had been genotyped as CYP2D6*34/*70. However, our HiFi data analysis for one of the South African participants revealed that the CYP2D6*2 SNP (rs16947, NG_008376.4:g.7870C>T, R296C) was in phase with the current CYP2D6*70 allele‐defining variants (**Figure** **5**). This participant had CYP2D6*5 (gene deletion) as the second allele in the diplotype.
**Case 2:** The second validated novel major allele (Haplotype 17, **Table** **4**) was also found in a South African participant with CYP2D6*5 as the second allele in the diplotype. HiFi sequencing showed that the rs141824015 missense variant (NG_008376.4:g.8206A>C, I339L) is in phase with the CYP2D6*41 defining variant rs28371725 (NG_008376.4: g.8008G>A, splice defect) on a *2 background (**Figure** **5**) in this haplotype.
**Suballeles**: Among the novel suballeles, we ascertained the presence of subvariants not yet cataloged by PharmVar for CYP2D6*149 and CYP2D6*36 (**Supplementary Materials** **S3**).

## DISCUSSION

African populations have been understudied for *CYP2D6* pharmacogenetic variation in comparison with European and Asian populations. To our knowledge, this study represents the most comprehensive investigation of *CYP2D6* genetic variation across SSA to date. We analyzed 961 high‐depth African genomes generated mainly by H3Africa projects, other collaborative projects within Africa, and the 1000 Genomes Project Consortium, for common, rare, and novel *CYP2D6* star alleles, including structural variants. In addition, we assessed the distribution of *CYP2D6* metabolizer phenotypes predicted from participant diplotypes across SSA, and performed laboratory validation for a subset of the novel star alleles via XL‐PCR and SMRT (Single‐Molecule Real‐Time) sequencing (Pacific Biosciences). Furthermore, we analyzed high coverage genomic data sets generated by the 1000 Genomes Project for comparison of the *CYP2D6* allele and phenotype distributions in other global biogeographical groups with the allelic landscape observed in SSA.

The known *CYP2D6* allele distributions in SSA populations are from studies among participants from Ethiopia, Kenya, Tanzania, Zimbabwe, South Africa, and Ghana.^14^, ^15^, ^16^, ^49^, ^50^, ^51^, ^52^, ^53^, ^54^ Most of these studies were relatively small‐scale by design, thus limiting the capacity for making allele distribution comparisons across diverse SSA populations and also for detecting novel alleles. In this study, we systematically present the *CYP2D6* allele distributions and predicted novel alleles from understudied populations for whom high‐coverage WGS data has recently been generated, e.g., the Berom in Nigeria, Fon in Benin, and participants from Burkina Faso, Botswana, Zambia, Cameroon, and South Africa.^20^, ^21^ In addition, our study included well‐studied populations (GWD, MSL, ESN, LWK, YRI) where previous lack of high‐coverage WGS data had made it virtually impossible to characterize the allelic variation in the hypervariable *CYP2D* region.^55^

Our *CYP2D6* star allele analysis reveals largely distinct allele distributions across the different SSA populations in this study, including differences between populations in neighboring countries and/or ethnolinguistic groups within the same country (e.g., the Berom, Esan, and Yoruba in Nigeria). However, the estimated frequencies of the rarer star alleles should be interpreted with caution given the relatively small sample sizes in the SSA populations included in this study. The frequencies of the key largely African‐specific decreased‐function *CYP2D6* alleles (i.e., *17 and *29) are consistent with previously reported frequencies from studies within SSA.^16^, ^17^, ^19^ However, they are by no means uniform across the whole of SSA—which is in keeping with the high genetic diversity across Africa. The frequencies of other key alleles such as CYP2D6*1, *41, and *5 in SSA were significantly different from the average frequency estimated by the PharmGKB from previous studies in the region (**Table** **2**). For CYP2D6*1 and *41, the frequency discrepancies could be due to differences in the data generation (e.g., WGS vs. genotyping) and/or star allele calling approaches across various studies. These star alleles (as well as *2) are common “backbone” alleles (i.e., their defining SNVs occur in multiple other haplotypes); hence their frequencies should be interpreted with caution especially from genotyping, panel‐based, or low‐coverage WGS analysis. The relatively high frequency (14–15%) of CYP2D6*5 (gene deletion) among southern African Bantu speakers is consistent with previously observed frequencies, for example among the Xhosa.^17^ Furthermore, some of the *CYP2D6* alleles, e.g., *4xN (no function), *36 (no function), *106 (uncertain function), *125 (unknown function), *1xN (increased function), and *2xN (increased function), not included in the SSA catalog in the current PharmGKB and CPIC *CYP2D6* reference materials were identified in SSA in this study. This is mostly due to the diverse sampling in our study and highlights the utility of high‐coverage WGS over allele‐specific genotyping, exome sequencing, and/or gene panel‐based approaches used in some of the previous SSA studies. A high prevalence (26%) of CYP2D6*2xN alleles has been reported among Ethiopian participants.^50^ However, it remains to be seen as to whether this relatively high CYP2D6*2xN frequency would be replicated using current orthogonal star allele characterization approaches^33^ and/or analysis of high‐coverage WGS data from Ethiopia. The added information about the distribution of *CYP2D6* star alleles across diverse African populations in our study will inform future research and precision medicine strategies such as pre‐emptive pharmacogenetic testing in clinical settings across Africa and globally.

There were some allele frequency differences observed between the SSA populations and the African American/Afro‐Caribbean participants included in the study. For example, CYP2D6*29 was found to be less frequent in the African American/Afro Caribbean populations than in SSA populations in the study, except for the FNB, GHA, and BOT participants where it was similar, while CYP2D6*4 (no function) was more frequent in the African African American/Afro Caribbean participants than in SSA. In line with previous pharmacogenomics studies in Africa,^21^, ^56^ our findings emphasize the fact that one African population should not be used as a general proxy for another, and certainly not for the whole of Africa, while developing personalized medicine implementation strategies. In addition, the differences in allele frequencies observed between SSA and European, East Asian, and South Asian populations underline the need for more comprehensive genotyping panels and/or sequencing approaches to assay the extensive *CYP2D6* allelic diversity in the different biogeographical groups.

We observed unique distributions of the CYP2D6 metabolizer phenotypes across different SSA populations and also in comparison with African American/Afro‐Caribbean, European, East Asian, and South Asian populations as a result of differences in the diplotype frequencies and in the distribution of key alleles. However, the relatively low sample sizes among the SSA populations in our study made it difficult to ascertain whether some of the observed differences in CYP2D6 phenotype distribution were statistically significant. We observed a relatively high proportion of SSA participants (over 10%) with indeterminate CYP2D6 metabolizer phenotype which exemplifies the limitations of the current CPIC guidelines for genotype–phenotype translation. These were mostly individuals carrying novel alleles or known alleles with uncertain function. This emphasizes the need for extensive allele characterization and phenotypic studies across Africa in order to develop effective precision medicine strategies on the continent, in particular for medications metabolized by CYP2D6.

Twenty‐seven potential novel *CYP2D6* star alleles were observed among the SSA populations included in this study given the high depth of the WGS data analyzed. The core variants defining these alleles were all rare and are not novel variants *per se*, but rather they are nonsynonymous variants that had either not been cataloged as allele‐defining by PharmVar at the time of our analysis or were found in different combinations in our study and evaluated based on the PharmVar criteria. The novel combinations of known core variants identified in our study represent allelic information that could be missed if comprehensive haplotyping is not done, e.g., when relying on genotyping arrays and imputation. In particular, rs368858603 (frameshift variant that introduces a stop codon on a *2 backbone) and rs536109057 (stop‐gain variant on a *29 backbone) have likely debilitating implications for the CPIC function of the relevant *CYP2D6* haplotypes. Further functional assays are needed before most of the novel star alleles predicted in this study are included in CPIC drug dosing guidelines. Given the genotyping and star allele calling challenges associated with *CYP2D6*, computationally inferred novel alleles should be interpreted with caution—especially for singletons.

Third‐generation sequencing approaches for characterizing star alleles in hypervariable pharmacogenes such as *CYP2D6* have been previously validated.^57^, ^58^, ^59^, ^60^ Through targeted HiFi long‐read sequencing (Pacific Biosciences), we characterized two of the novel *CYP2D6* star alleles and also found multiple novel suballeles. The process of submitting these novel alleles to PharmVar is ongoing. Furthermore, validation of predicted novel *CYP2D6* star alleles occurring in the SSA participants with corresponding DNA samples available from the Coriell Institute (Camden) is already underway as part of an ongoing collaboration with three labs led by PharmVar Consortium members.^7^

Our study had some limitations. Firstly, we focused our analysis on SSA and did not analyze any northern African genomes. We also had a limited number of samples from speakers of non‐Bantu languages in SSA, such as the Khoe and San. Secondly, we were unable to computationally resolve 13 *CYP2D6* diplotypes in SSA either due to presence of novel core variants that could not be phased or due to potential uncharacterized structural variations that were novel to all the algorithms used in the star allele calling. Coriell sample IDs of participants with these diplotypes have been summarized in **Supplementary Materials** **S3** as they could be useful in follow‐up studies to this work. Thirdly, *in vitro* and *in vivo* function studies were out of the scope of this work, thus limiting our assessment of the impact of star alleles with unknown function and novel alleles on CYP2D6‐mediated drug metabolism. The variant deleteriousness predictions by the VEP plugins used in the study may not necessarily correlate with the CPIC clinical function and they are difficult to apply when examining the functional impact of haplotypes. Future studies employing *in vitro* assays such as deep mutational scans^61^ and/or *in vivo* assays are therefore warranted to investigate the impact of alleles with unknown or uncertain function on CYP2D6‐mediated drug metabolism. Regarding phenotype prediction in general, there are a number of factors that could influence the CYP2D6 phenotype such as phenoconversion, which we did not investigate due to insufficient metadata. Considering that there is variability in geography‐associated selection pressures across Africa, gene–environment interactions could also potentially influence the star allele clinical function and treatment outcomes in general. Fourthly, in our laboratory validation, *CYP2D6* XL‐PCR products for 48 participants failed to barcode during the HiFi sequencing library preparation due to technical challenges that arose from pooling the *CYP2D6* amplicons with those from *CYP2A6* and *CYP2B6*. We mitigated this by doing separate barcoding for select amplicons from samples that had predicted novel *CYP2D6* star alleles or suballeles.

In conclusion, this study has revealed key insights regarding the *CYP2D6* pharmacogenetic variation mainly in SSA populations. Our results emphasize the need for precision medicine across Africa and within the African diaspora to ensure safety and efficacy of treatments, particularly those metabolized by CYP2D6. The relatively high number of potential novel *CYP2D6* star alleles observed in SSA highlights the need for in‐depth pharmacogene star allele characterization across Africa, especially in understudied ethnolinguistic groups. We recommend the consensus *CYP2D6* star allele calling approach used in this study for similar analyses focusing on African and other global populations especially when researchers and clinical geneticists have high‐depth WGS data available to them. Future work entailing the unequivocal characterization of the novel alleles not validated in this study as well as functional studies to determine their clinical implications will be critical in supporting clinical pharmacogenomics implementation strategies across Africa and in global settings.

## FUNDING

This study was funded by a grant from GlaxoSmithKline R&D Ltd. (GSK) to the Wits Health Consortium. GSK had no role in the study design, data collection and analysis. D.T. was partially supported by funding from the South African National Research Foundation (NRF grant number: 128895). The whole genome sequencing of the Human Heredity and Health in Africa (H3Africa) Data was supported by a grant from the National Human Genome Research Institute, National Institutes of Health (NIH/NHGRI, Grant U54HG003273). The AWI‐Gen Collaborative Center is funded by the NIH/NHGRI (Grant U54HG006938) as part of the H3Africa Consortium. M.R. is a South African Research Chair in Genomics and Bioinformatics of African Populations hosted by the University of the Witwatersrand, funded by the Department of Science and Technology (South Africa), and administered by National Research Foundation of South Africa (NRF). The TrypanoGEN project was funded by the Wellcome Trust (study number 099310/Z/12/Z). The Collaborative African Genetics Network (CAfGEN) is funded by the NIH/NHGRI (Grant 1U54AI110398). The African Collaborative Center for Microbiome and Genomics Research is funded by the NIH/NHGRI (Grant U54HG006947). The primary work relating to DNA sample processing and WGS at the Cell Biology Research Lab is based on research supported by grant awards from the Strategic Health Innovation Partnerships (SHIP) Unit of the South African Medical Research Council, a grantee of the Bill and Melinda Gates Foundation, and the South African Research Chairs Initiative of the Department of Science and Technology and National Research Foundation of South Africa (84177). The opinions, findings, and conclusions or recommendations expressed in this manuscript are solely the responsibility of the authors and not necessarily to be attributed to GSK, NRF, Wellcome Trust, or the NIH/NHGRI.

## CONFLICT OF INTEREST

The authors declared no competing interests for this work.

## AUTHOR CONTRIBUTIONS

D.T., B.I.D., G.E.B.W., M.P., G.A., P.R.B., C.A., M.M., G.S., M.C.S., C.T.T., M.R., Z.L., and S.H. wrote the manuscript. D.T., B.I.D., G.E.B.W., Z.L., and S.H. designed the research. D.T., Z.L., and S.H. performed the research. D.T. analyzed the data. C.A., M.M., G.S., M.C.S., C.T.T., and M.R. contributed new study data sets and materials.

## Supporting information


Note S1

Material S2

Material S3

## Data Availability

The 1000 Genomes data and the SGDP data are publicly available. The H3Africa data sets used in this study are available from European Genome‐Phenome Archive (https://ega‐archive.org/) on application to the relevant Data Access Committees (EGADs: EGAD00001003791, EGAD00001006418, EGAD00001004220, EGAD00001004448, EGAD00001004505, EGAD00001004533, EGAD00001004557, EGAD00001004393, EGAD00001007589).

## References

[1] Whirl‐Carrillo, M. *et al*. An evidence‐based framework for evaluating pharmacogenomics knowledge for personalized medicine. Clin. Pharmacol. Ther. 110, 563–572 (2021). 34216021 10.1002/cpt.2350 PMC8457105

[2] Scott, S.A. Personalizing medicine with clinical pharmacogenetics. Genet. Med. 13, 987–995 (2011). 22095251 10.1097/GIM.0b013e318238b38c PMC3290900

[3] Zanger, U.M. & Schwab, M. Cytochrome P450 enzymes in drug metabolism: regulation of gene expression, enzyme activities, and impact of genetic variation. Pharmacol. Ther. 138, 103–141 (2013). 23333322 10.1016/j.pharmthera.2012.12.007

[4] Wright, G.E.B. , Carleton, B. , Hayden, M.R. & Ross, C.J.D. The global spectrum of protein‐coding pharmacogenomic diversity. Pharmacogenomics J. 18, 187–195 (2018). 27779249 10.1038/tpj.2016.77 PMC5817389

[5] Gaedigk, A. , Sangkuhl, K. , Whirl‐Carrillo, M. , Klein, T. & Leeder, J.S. Prediction of CYP2D6 phenotype from genotype across world populations. Genet. Med. 19, 69–76 (2017). 27388693 10.1038/gim.2016.80 PMC5292679

[6] Zhou, Y. , Ingelman‐Sundberg, M. & Lauschke, V.M. Worldwide distribution of cytochrome P450 alleles: a meta‐analysis of population‐scale sequencing projects. Clin. Pharmacol. Ther. 102, 688–700 (2017). 28378927 10.1002/cpt.690 PMC5600063

[7] Gaedigk, A. , Casey, S.T. , Whirl‐Carrillo, M. , Miller, N.A. & Klein, T.E. Pharmacogene variation consortium: a global resource and repository for Pharmacogene variation. Clin. Pharmacol. Ther. 110, 542–545 (2021). 34091888 10.1002/cpt.2321 PMC8725060

[8] Caudle, K.E. *et al*. Standardizing *CYP2D6* genotype to phenotype translation: consensus recommendations from the clinical pharmacogenetics implementation consortium and Dutch pharmacogenetics working group. Clin. Transl. Sci. 13, 116–124 (2020). 31647186 10.1111/cts.12692 PMC6951851

[9] Kimura, S. , Umeno, M. , Skoda, R.C. , Meyer, U.A. & Gonzalez, F.J. The human debrisoquine 4‐hydroxylase (CYP2D) locus: sequence and identification of the polymorphic CYP2D6 gene, a related gene, and a pseudogene. Am. J. Hum. Genet. 45, 889–904 (1989). 2574001 PMC1683468

[10] Nofziger, C. *et al*. PharmVar GeneFocus: *CYP2D6* . Clin. Pharmacol. Ther. 107, 154–170 (2020). 31544239 10.1002/cpt.1643 PMC6925641

[11] Yang, Y. , Botton, M.R. , Scott, E.R. & Scott, S.A. Sequencing the *CYP2D6* gene: from variant allele discovery to clinical pharmacogenetic testing. Pharmacogenomics 18, 673–685 (2017). 28470112 10.2217/pgs-2017-0033 PMC5591463

[12] Turner, A.J. *et al*. Identification of *CYP2D6* haplotypes that interfere with commonly used assays for copy number variation characterization. J. Mol. Diagn. 23, 577–588 (2021). 33631352 10.1016/j.jmoldx.2021.01.013 PMC8176139

[13] Nofziger, C. & Paulmichl, M. Accurately genotyping *CYP2D6*: not for the faint of heart. Pharmacogenomics 19, 999–1002 (2018). 30020016 10.2217/pgs-2018-0105

[14] Masimirembwa, C. , Persson, I. , Bertilsson, L. , Hasler, J. & Ingelman‐Sundberg, M. A novel mutant variant of the *CYP2D6* gene (CYP2D6*17) common in a black African population: association with diminished debrisoquine hydroxylase activity. Br. J. Clin. Pharmacol. 42, 713–719 (1996). 8971426 10.1046/j.1365-2125.1996.00489.x PMC2042718

[15] Wennerholm, A. *et al*. Characterization of the CYP2D6*29 allele commonly present in a Black Tanzanian population causing reduced catalytic activity. Pharmacogenetics 11, 417–427 (2001). 11470994 10.1097/00008571-200107000-00005

[16] Matimba, A. , Del‐Favero, J. , Van Broeckhoven, C. & Masimirembwa, C. Novel variants of major drug‐metabolising enzyme genes in diverse African populations and their predicted functional effects. Hum. Genomics 3, 169–190 (2009). 19164093 10.1186/1479-7364-3-2-169 PMC3525272

[17] Wright, G.E.B. *et al*. Elucidation of CYP2D6 genetic diversity in a unique African population: implications for the future application of pharmacogenetics in the Xhosa population. Ann. Hum. Genet. 74, 340–350 (2010). 20597905 10.1111/j.1469-1809.2010.00585.x

[18] Dodgen, T.M. *et al*. Risperidone‐associated adverse drug reactions and CYP2D6 polymorphisms in a south African cohort. Appl Transl Genom 5, 40–46 (2015). 26937359 10.1016/j.atg.2015.05.001 PMC4745369

[19] Dodgen, T.M. *et al*. Introduction of the AmpliChip CYP450 test to a South African cohort: a platform comparative prospective cohort study. BMC Med. Genet. 14, 20 (2013). 23356658 10.1186/1471-2350-14-20 PMC3605304

[20] Choudhury, A. *et al*. High‐depth African genomes inform human migration and health. Nature 586, 741–748 (2020). 33116287 10.1038/s41586-020-2859-7 PMC7759466

[21] da Rocha, J.E.B. *et al*. The extent and impact of variation in ADME genes in sub‐Saharan African populations. Front. Pharmacol. 12, 634016 (2021). 34721006 10.3389/fphar.2021.634016 PMC8549571

[22] Sengupta, D. *et al*. Genetic substructure and complex demographic history of South African Bantu speakers. Nat. Commun. 12, 2080 (2021). 33828095 10.1038/s41467-021-22207-y PMC8027885

[23] Twesigomwe, D. *et al*. StellarPGx: a Nextflow pipeline for calling star alleles in cytochrome P450 genes. Clin. Pharmacol. Ther. 110, 741–749 (2021). 33492672 10.1002/cpt.2173

[24] Numanagić, I. *et al*. Allelic decomposition and exact genotyping of highly polymorphic and structurally variant genes. Nat. Commun. 9, 828 (2018). 29483503 10.1038/s41467-018-03273-1 PMC5826927

[25] Lee, S.‐B. , Wheeler, M.M. , Thummel, K.E. & Nickerson, D.A. Calling star alleles with stargazer in 28 Pharmacogenes with whole genome sequences. Clin. Pharmacol. Ther. 106, 1328–1337 (2019). 31206625 10.1002/cpt.1552 PMC6896231

[26] Chen, X. *et al*. Cyrius: accurate *CYP2D6* genotyping using whole‐genome sequencing data. Pharmacogenomics J. 21, 251–261 (2021). 33462347 10.1038/s41397-020-00205-5 PMC7997805

[27] Twist, G.P. *et al*. Constellation: a tool for rapid, automated phenotype assignment of a highly polymorphic pharmacogene, *CYP2D6*, from whole‐genome sequences. NPJ Genom. Med. 1, 15007 (2016). 29263805 10.1038/npjgenmed.2015.7 PMC5685293

[28] Choudhury, A. *et al*. Whole‐genome sequencing for an enhanced understanding of genetic variation among South Africans. Nat. Commun. 8, 2062 (2017). 29233967 10.1038/s41467-017-00663-9 PMC5727231

[29] Mallick, S. *et al*. The Simons genome diversity project: 300 genomes from 142 diverse populations. Nature 538, 201–206 (2016). 27654912 10.1038/nature18964 PMC5161557

[30] Byrska‐Bishop, M. *et al*. High‐coverage whole‐genome sequencing of the expanded 1000 genomes project cohort including 602 trios. Cell 185, 3426–3440.e19 (2022). 36055201 10.1016/j.cell.2022.08.004 PMC9439720

[31] Li, H. *et al*. The sequence alignment/map format and SAMtools. Bioinformatics 25, 2078–2079 (2009). 19505943 10.1093/bioinformatics/btp352 PMC2723002

[32] Robinson, J.T. , Thorvaldsdóttir, H. , Wenger, A.M. , Zehir, A. & Mesirov, J.P. Variant review with the integrative genomics viewer. Cancer Res. 77, e31–e34 (2017). 29092934 10.1158/0008-5472.CAN-17-0337 PMC5678989

[33] Gaedigk, A. *et al*. Characterization of reference materials for genetic testing of *CYP2D6* alleles: a GeT‐RM collaborative project. J. Mol. Diagn. 21, 1034–1052 (2019). 31401124 10.1016/j.jmoldx.2019.06.007 PMC6854474

[34] Gaedigk, A. *et al*. Cytochrome P4502D6 (*CYP2D6*) gene locus heterogeneity: characterization of gene duplication events. Clin. Pharmacol. Ther. 81, 242–251 (2007). 17259947 10.1038/sj.clpt.6100033

[35] Breese, M.R. & Liu, Y. NGSUtils: a software suite for analyzing and manipulating next‐generation sequencing datasets. Bioinformatics 29, 494–496 (2013). 23314324 10.1093/bioinformatics/bts731 PMC3570212

[36] Poplin, R. *et al*. A universal SNP and small‐indel variant caller using deep neural networks. Nat. Biotechnol. 36, 983–987 (2018). 30247488 10.1038/nbt.4235

[37] Patterson, M. *et al*. WhatsHap: weighted haplotype assembly for future‐generation sequencing reads. J. Comput. Biol. 22, 498–509 (2015). 25658651 10.1089/cmb.2014.0157

[38] McLaren, W. *et al*. The Ensembl variant effect predictor. Genome Biol. 17, 122 (2016). 27268795 10.1186/s13059-016-0974-4 PMC4893825

[39] Kumar, P. , Henikoff, S. & Ng, P.C. Predicting the effects of coding non‐synonymous variants on protein function using the SIFT algorithm. Nat. Protoc. 4, 1073–1081 (2009). 19561590 10.1038/nprot.2009.86

[40] Adzhubei, I. , Jordan, D.M. & Sunyaev, S.R. Predicting functional effect of human missense mutations using PolyPhen‐2. Curr. Protoc. Hum. Genet. Chapter 7, Unit7.20 (2013). 10.1002/0471142905.hg0720s76 PMC4480630 23315928

[41] Rentzsch, P. , Witten, D. , Cooper, G.M. , Shendure, J. & Kircher, M. CADD: predicting the deleteriousness of variants throughout the human genome. Nucleic Acids Res. 47, D886–D894 (2019). 30371827 10.1093/nar/gky1016 PMC6323892

[42] Chun, S. & Fay, J.C. Identification of deleterious mutations within three human genomes. Genome Res. 19, 1553–1561 (2009). 19602639 10.1101/gr.092619.109 PMC2752137

[43] Choi, Y. , Sims, G.E. , Murphy, S. , Miller, J.R. & Chan, A.P. Predicting the functional effect of amino acid substitutions and indels. PLoS One 7, e46688 (2012). 23056405 10.1371/journal.pone.0046688 PMC3466303

[44] Carter, H. , Douville, C. , Stenson, P.D. , Cooper, D.N. & Karchin, R. Identifying Mendelian disease genes with the variant effect scoring tool. BMC Genomics 14(Suppl 3), S3 (2013). 10.1186/1471-2164-14-S3-S3 PMC3665549 23819870

[45] Zhou, Y. , Mkrtchian, S. , Kumondai, M. , Hiratsuka, M. & Lauschke, V.M. An optimized prediction framework to assess the functional impact of pharmacogenetic variants. Pharmacogenomics J. 19, 115–126 (2019). 30206299 10.1038/s41397-018-0044-2 PMC6462826

[46] Hu, J. & Ng, P.C. SIFT indel: predictions for the functional effects of amino acid insertions/deletions in proteins. PLoS One 8, e77940 (2013). 24194902 10.1371/journal.pone.0077940 PMC3806772

[47] Karczewski, K.J. *et al*. The mutational constraint spectrum quantified from variation in 141,456 humans. Nature 581, 434–443 (2020). 32461654 10.1038/s41586-020-2308-7 PMC7334197

[48] Byrska‐Bishop, M. *et al*. High coverage whole genome sequencing of the expanded 1000 genomes project cohort including 602 trios. *bioRxiv* 2021.02.06.430068 (2021) 10.1101/2021.02.06.430068 PMC9439720 36055201

[49] Ahmed, J.H. *et al*. CYP2D6 genotype predicts plasma concentrations of tamoxifen metabolites in Ethiopian breast cancer patients. Cancers (Basel) 11, 1353 (2019). 31547390 10.3390/cancers11091353 PMC6770728

[50] Aklillu, E. *et al*. Frequent distribution of ultrarapid metabolizers of debrisoquine in an Ethiopian population carrying duplicated and multiduplicated functional CYP2D6 alleles. J. Pharmacol. Exp. Ther. 278, 441–446 (1996). 8764380

[51] Bathum, L. *et al*. Phenotypes and genotypes for CYP2D6 and CYP2C19 in a black Tanzanian population. Br. J. Clin. Pharmacol. 48, 395–401 (1999). 10510152 10.1046/j.1365-2125.1999.00019.x PMC2014329

[52] Dandara, C. *et al*. Genetic polymorphism of CYP2D6 and CYP2C19 in east‐ and southern African populations including psychiatric patients. Eur. J. Clin. Pharmacol. 57, 11–17 (2001). 11372584 10.1007/s002280100282

[53] Gutiérrez Rico, E.M. *et al*. CYP2D6 genotyping analysis and functional characterization of novel allelic variants in a Ni‐Vanuatu and Kenyan population by assessing dextromethorphan O‐demethylation activity. Drug Metab. Pharmacokinet. 35, 89–101 (2020). 32037159 10.1016/j.dmpk.2019.07.003

[54] Griese, E.U. , Asante‐Poku, S. , Ofori‐Adjei, D. , Mikus, G. & Eichelbaum, M. Analysis of the CYP2D6 gene mutations and their consequences for enzyme function in a west African population. Pharmacogenetics 9, 715–723 (1999). 10634134

[55] Drögemöller, B.I. , Wright, G.E.B. , Niehaus, D.J.H. , Emsley, R. & Warnich, L. Next‐generation sequencing of pharmacogenes: a critical analysis focusing on schizophrenia treatment. Pharmacogenet. Genomics 23, 666–674 (2013). 24141736 10.1097/FPC.0000000000000006

[56] Rajman, I. , Knapp, L. , Morgan, T. & Masimirembwa, C. African genetic diversity: implications for cytochrome P450‐mediated drug metabolism and drug development. EBioMedicine 17, 67–74 (2017). 28237373 10.1016/j.ebiom.2017.02.017 PMC5360579

[57] Ammar, R. , Paton, T.A. , Torti, D. , Shlien, A. & Bader, G.D. Long read nanopore sequencing for detection of *HLA* and *CYP2D6* variants and haplotypes. F1000Res 4, 17 (2015). 25901276 10.12688/f1000research.6037.1 PMC4392832

[58] Buermans, H.P.J. *et al*. Flexible and scalable full‐length CYP2D6 long amplicon PacBio sequencing. Hum. Mutat. 38, 310–316 (2017). 28044414 10.1002/humu.23166 PMC5324676

[59] Liau, Y. *et al*. Nanopore sequencing of the pharmacogene CYP2D6 allows simultaneous haplotyping and detection of duplications. Pharmacogenomics 20, 1033–1047 (2019). 31559921 10.2217/pgs-2019-0080

[60] Qiao, W. *et al*. Long‐read single molecule real‐time full gene sequencing of cytochrome P450‐2D6. Hum. Mutat. 37, 315–323 (2016). 26602992 10.1002/humu.22936 PMC4752389

[61] Amorosi, C.J. *et al*. Massively parallel characterization of CYP2C9 variant enzyme activity and abundance. Am. J. Hum. Genet. 108, 1735–1751 (2021). 34314704 10.1016/j.ajhg.2021.07.001 PMC8456167

