# Supplementary material for: Characterization of CYP2D6 Pharmacogenetic Variation in Sub‐Saharan African Populations
Source: Clin Pharmacol Ther. 2022 Oct 21;113(3):643–59. doi: 10.1002/cpt.2749 (PMC9957841; doi:10.1002/cpt.2749)
Supplement: Supplementary file 1 — Note S1 [file CPT-113-643-s003.pdf]

Characterisation of *CYP2D6* pharmacogenetic variation in sub-Saharan African populations

Supplementary note

Local ethics approvals for participating studies

The H3Africa AWI-Gen study (provided data from Ghana, Burkina Faso and South Africa) was approved by the University of the Witwatersrand Human Research Ethics Committee (HREC Medical) under protocol numbers M121029 and M170880. The (South Africa) data from the Cell Biology Research Laboratory (CBRL), National Institute for Communicable Diseases (NICD, Wits) was generated by a study approved by the Wits HREC Medical (protocol number M140926). The SAHGP study was approved by the Wits HREC Medical (protocol number M120223). The H3Africa Benin study was approved by the Comité d'éthique de la recherche, Université de Montréal. The H3Africa CAfGEN study (provided data from Botswana) was approved by the Institutional Review Board (IRB) of the Ministry of Health of the Republic of Botswana (PPPME-13/18/1). The H3Africa TrypanoGEN Study (Cameroon component) was approved by the Comité National D'Ethique de la Recherche pour la Santé Humaine of the Republic of Cameroon (2013/11/364/L/CNERSH/SP). The H3Africa TrypanoGEN Study (Zambian component) was approved by the Biomedical Research Ethics Committee of the University of Zambia (FWA00000338). The H3Africa ACCME Study (Nigeria) was approved by the National Health Research Ethics Committee of Nigeria (NHREC/01/01/2007-29/11/2016).
