# Supplementary material for: Characterization of CYP2D6 Pharmacogenetic Variation in Sub‐Saharan African Populations
Source: Clin Pharmacol Ther. 2022 Oct 21;113(3):643–59. doi: 10.1002/cpt.2749 (PMC9957841; doi:10.1002/cpt.2749)
Supplement: Supplementary file 2 — Material S2 [file CPT-113-643-s002.pdf]

Characterisation of *CYP2D6* pharmacogenetic variation in sub-Saharan African populations

Supplementary Materials

Figure S1: Overview of the *CYP2D6* star allele calling approach used in the study

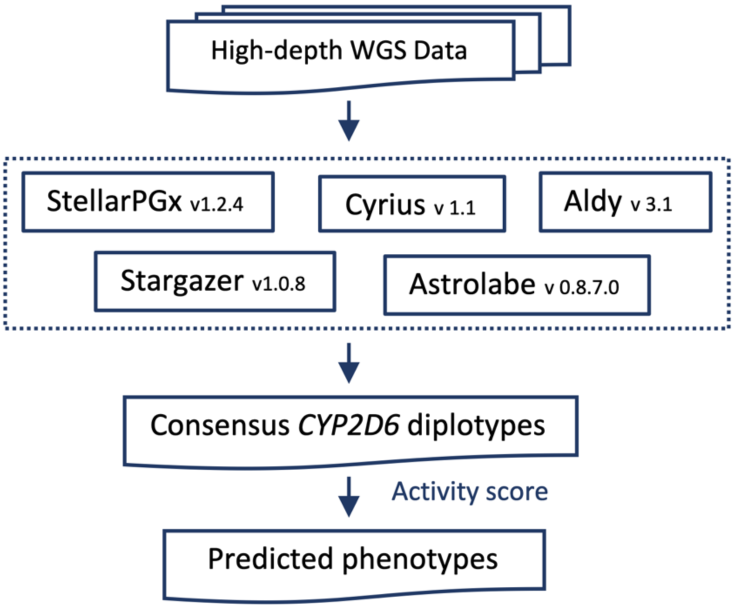

StellarPGx, Cyrius, and Aldy required only the Binary Alignment (BAM) file as input and called sample diplotypes from data aligned to both the GRCh37 and GRCh38 reference genomes. For Astrolabe and Stargazer, the required Variant Call Format (VCF) files were generated using the H3ABioNet's GATK pipeline (https://github.com/h3abionet/recalling). Briefly gVCFs were called using HaplotypeCaller and then combined using CombineGVCF in GATK v4.0.8.1. Raw variants were joint-called using GenotypeGVCFs (v4.1.3.0). The SNPs and indels in the WGS VCFs were subjected to Variant Quality Score Recalibration, and thereafter the high quality (PASS) variants corresponding to the CYP2D6 loci were extracted from the WGS VCF using BCFtools. Astrolabe and Stargazer also required the BAM file and 'GATK-DepthOfCoverage Format' (GDF) files, respectively, for depth of coverage analysis and structural variant calling. The *VDR* gene was used as the control locus for Stargazer's star allele analysis.

Note: Stargazer v1.0.8 does not have support for GRCh38. The GRCh38-aligned whole genome sequence (WGS) data included in this study was the 1000 Genomes WGS data. In order to run Stargazer on this data, we extracted reads aligned to the CYP2D, and VDR (control gene locus) GRCh38 regions using SAMtools, and realigned them to GRCh37 using BWA-MEM. The variant calling and depth of coverage analysis was similar to the aforementioned steps for the WGS data except that we performed hard-filtering in the variant quality control for the realigned regional data. Stargazer was then run on the CYP2D6 VCF files and the required GDF files for these samples.

CYP2D6 long-range PCR

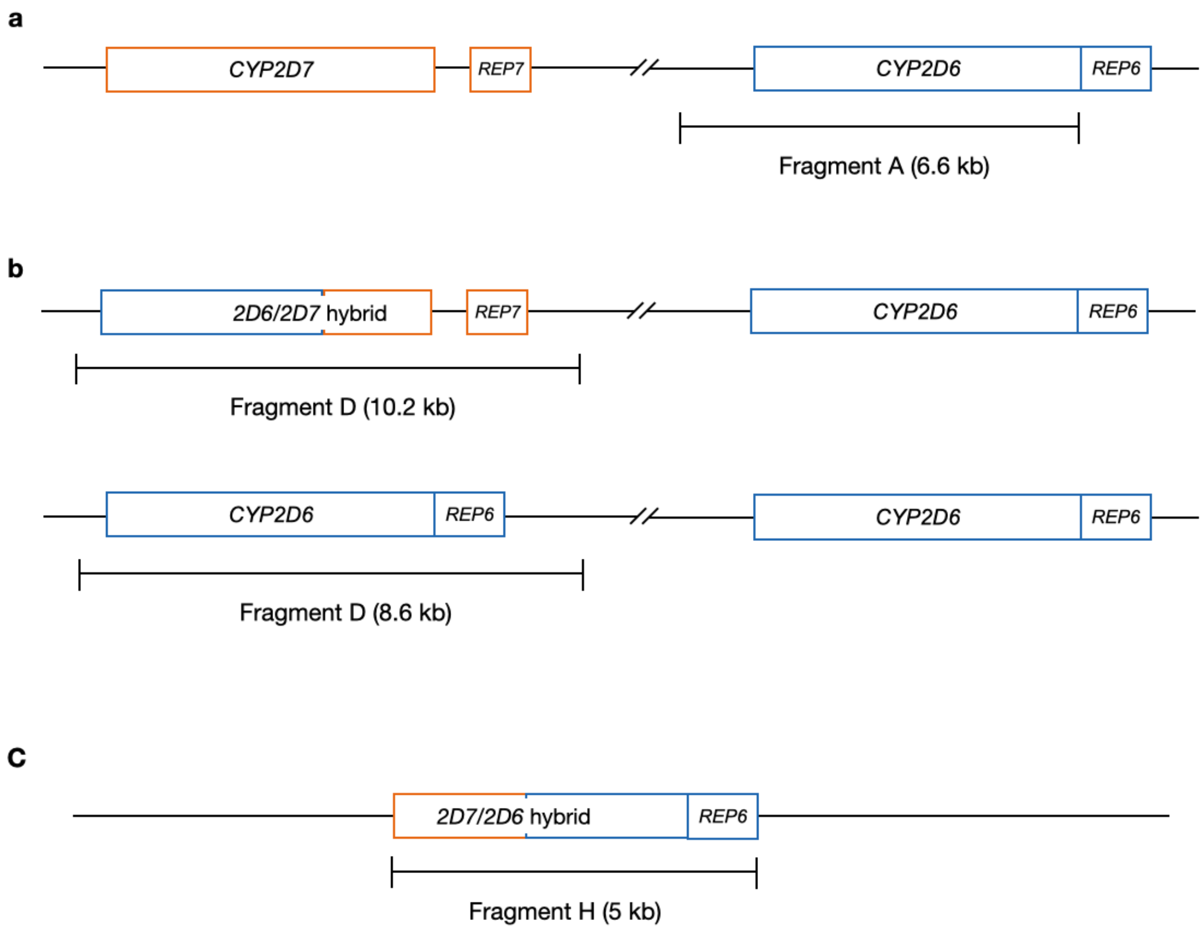

Figure S2: *CYP2D6* XL-PCR fragments generated for SMRT sequencing. (a) Depicts the arrangement without structural variations (except for the intron 1 conversion in some cases). (b) Depicts Fragment D (FragD) which may arise due to duplication of a normal *CYP2D6* copy (8.6 kb fragment) or due to *CYP2D6-2D7* hybrid formation (10.2 kb fragment). (c) Graphical representation of a *CYP2D7-2D6* hybrid fragment (*CYP2D6*13* alleles).

Table S1: Primers used for generating *CYP2D6* long-range PCR fragments in this study

Assay	Primer name	Direction	5'–sequence	References
CYP2D6 FragA (6.6 kb)	5'2D6 6F	F	TCACCCCCAGCGGACTTATCAACC	(Gaedigk et al., 2007)
	3'2D6 R/3	R	CGACTGAGCCCTGGGAGGTAGGTAG	(Gaedigk et al., 2007)
CYP2D6 FragD (8.6 kb or 10.2 kb)	5'2D6 F/2	F	CCAGAAGGCTTTGCAGGCTTCAG	(Gaedigk et al., 2007)
	3'2D6x2 R/3	R	CGGCAGTGGTCAGCTAATGAC	(Gaedigk et al., 2007)
CYP2D6 FragH (~5 kb)	5'2D7 XL-5F	F	TCCGACCAGGCCTTTCTACCAC	(Gaedigk and Coetsee, 2008)
	3'2D6 R/3	R	CGACTGAGCCCTGGGAGGTAGGTAG	(Gaedigk and Coetsee, 2008)

F, Forward; R, Reverse

Table S2: PCR cycling conditions for generating *CYP2D6* fragments (first amplification)

	CYP2D6 assays		
	1	2	3
First denaturation (ºC:s)	94:180	94:180	94:180
Denaturation (ºC:s)	94:20	94:20	94:20
Annealing (ºC:s)	68:30	68:30	68:30
Extension (ºC:s)	68:420	68:780	68:420
Extension (ºC:s)	68:420	68:780	68:420
Final extension (ºC:s)	68:420	68:780	68:420
Hold	10ºC	10ºC	10ºC

[1]Amplification for *CYP2D6* FragA.

[2]Amplification for *CYP2D6* FragD.

[3]Amplification for *CYP2D6* FragH.

References

Gaedigk, A. *et al.* (2007). Cytochrome P450 2D6 (*CYP2D6*) gene locus heterogeneity: characterization of gene duplication events. *Clinical Pharmacology and Therapeutics* **81**, 242–251.

Gaedigk, A., and Coetsee, C. (2008). The *CYP2D6* gene locus in South African Coloureds: unique allele distributions, novel alleles and gene arrangements. *European Journal of Clinical Pharmacology*, 64(5), 465–475.
