# Supplementary material for: Characterization of CYP2D6 Pharmacogenetic Variation in Sub‐Saharan African Populations
Source: Clin Pharmacol Ther. 2022 Oct 21;113(3):643–59. doi: 10.1002/cpt.2749 (PMC9957841; doi:10.1002/cpt.2749)
Supplement: Supplementary file 3 — Material S3 [file CPT-113-643-s001.pdf]

**Characterisation of *CYP2D6* pharmacogenetic variation in sub-Saharan African populations**

**Supplementary Materials**

**Figure S3**: Proportion of *CYP2D6* star alleles defined by structural variants (270 alleles) and core SNVs (1598 alleles) in sub-Saharan African populations

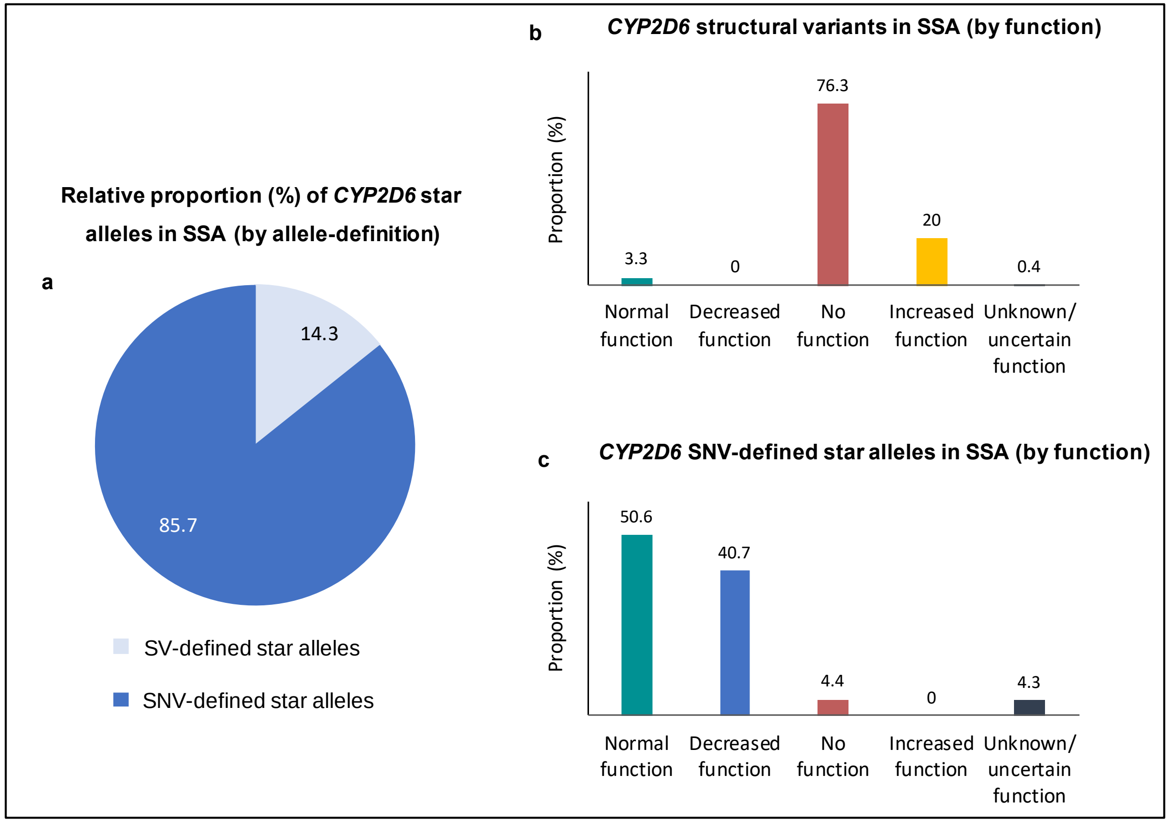


Panel (**a**) shows the relative proportion *CYP2D6* star alleles defined by structural variations (SVs) — e.g. the gene deletion (**5*), gene duplications, hybrids and tandems — compared to the relative proportion alleles defined by various combinations of key SNVs including rs28371706 (**17*), rs61736512 and rs59421388 (**29*), and rs1065852 (**10*). Panel (**b**) depicts the relative proportion of structural variants by function across SSA. *CYP2D6*5* accounts for the high proportion of no function alleles. Normal function SV-defined alleles arise from duplication of decreased function SNV-defined alleles e.g. *CYP2D6*17x2* and **29x2*, while increased function SV-defined alleles mainly include duplications of normal function alleles e.g. *CYP2D6*1xN* and **2xN*. Panel (**c**) shows the relative proportions of SNV-defined alleles grouped by CPIC function. *CYP2D6*17* and **29* account for the high proportion of decreased function alleles across SSA.

**Table S3:** Full table showing frequencies of all *CYP2D6* star alleles called in all populations in this study (sub-Saharan African and global populations)

| | | Allele frequencies (%) | | | | | | |
|---|---|---|---|---|---|---|---|---|
| ***CYP2D6* allele** | **CPIC function** | **SSA in this study (n=947)** | **Previous SSA studies[a] (n=2248)** | **African American/ Afro-Caribbean (n=157)** | **EUR (n=498)** | **AMR (n=345)** | **SAS (n=481)** | **EAS (n=502)** |
| *1 | Normal | 24.7 | 7.8 | 31.5 | 35.8 | 45.1 | 39.5 | 26 |
| *2 | Normal | 13.4 | 19.8 | 9.6 | 15.9 | 18.3 | 20.8 | 7.8 |
| *13+*1 | Normal | 0 | | 0 | 0.1 | 0 | 0 | 0 |
| *13+*2 | Normal | 0 | | 0 | 0.2 | 0.4 | 0.1 | 0 |
| *27 | Normal | 0.3 | 0.5 | 0.3 | 0 | 0.3 | 0 | 0 |
| *33 | Normal | 0 | | 0 | 0.6 | 0.3 | 0.2 | 0 |
| *34 | Normal | 0.1 | | 0 | 0 | 0 | 0 | 0 |
| *35 | Normal | 0 | 0 | 1 | 4.8 | 2.6 | 0.6 | 0 |
| *39 | Normal | 0.1 | 0 | 0.3 | 0.1 | 0.1 | 0.4 | 0.3 |
| *45 | Normal | 3.7 | 4.2 | 2.2 | 0 | 0.3 | 0 | 0 |
| *46 | Normal | 0.5 | 0.2 | 1 | 0 | 0.1 | 0 | 0 |
| *17x2 | Normal | 0.2 | | 0 | 0 | 0 | 0 | 0 |
| *29x2 | Normal | 0.3 | | 0.3 | 0 | 0 | 0 | 0 |
| *9x2 | Normal | 0 | | 0 | 0.1 | 0 | 0 | 0 |
| *41x2 | Normal | 0 | 0.2 | 0 | 0 | 0 | 0 | 0 |
| *68+*2 | Normal | 0 | | 0 | 0.1 | 0 | 0.1 | 0 |
| *83+*2 | Normal | 0 | | 0 | 0 | 0.1 | 0 | 0 |
| *9 | Decreased | 0 | 0 | 0.3 | 2.4 | 1.3 | 0 | 0 |
| *10 | Decreased | 3.9 | 5.6 | 3.8 | 1.2 | 1.6 | 3.5 | 15.2 |
| *10x2 | Decreased | 0 | 0 | 0 | 0 | 0 | 0 | 0.4 |
| *14 | Decreased | 0 | | 0 | 0 | 0 | 0 | 1 |
| *17 | Decreased | 19.5 | 19.3 | 16.9 | 0.2 | 0.9 | 0 | 0 |
| *29 | Decreased | 10 | 12.1 | 5.7 | 0 | 0.3 | 0 | 0 |

| | | Allele frequencies (%) | | | | | | |
|---|---|---|---|---|---|---|---|---|
| *CYP2D6* allele | CPIC function | SSA in this study (n=947) | Previous SSA studies[a] (n=2248) | African American/ Afro-Caribbean (n=157) | EUR (n=498) | AMR (n=345) | SAS (n=481) | EAS (n=502) |
| **36+*10* | Decreased | 0 | | 0.3 | 0 | 0.1 | 1.2 | 34.5 |
| **36+*10.003* | Decreased | 0 | | 0 | 0 | 0 | 0 | 0.1 |
| **36x2+*10* | Decreased | 0 | | 0 | 0 | 0 | 0 | 1.7 |
| **36+*10x2* | Decreased | 0 | | 0 | 0 | 0 | 0 | 0.5 |
| **36+*10≥3* | Decreased | 0 | | 0 | 0 | 0 | 0 | 0.1 |
| **36x2+*10x2* | Decreased | 0 | | 0 | 0 | 0 | 0 | 0.1 |
| **36x2+*10≥3* | Decreased | 0 | | 0 | 0 | 0 | 0 | 0.1 |
| **41* | Decreased | 0.8 | 11.5 | 2.9 | 8.5 | 6.1 | 11.4 | 3.4 |
| **49* | Decreased | 0 | 0 | 0 | 0 | 0 | 0 | 0.5 |
| **59* | Decreased | 0 | | 0 | 0.2 | 0.1 | 0 | 0 |
| **84* | Decreased | 0.1 | | 0.3 | 0 | 0 | 0 | 0 |
| **3* | No function | 0 | 0.2 | 1 | 1.8 | 0.6 | 0.2 | 0 |
| **4* | No function | 1.7 | 3.4 | 4.1 | 11.8 | 9.6 | 8.2 | 0.2 |
| **4x2* | No function | 2.2 | | 3.8 | 0.3 | 0.1 | 0 | 0 |
| **4x3* | No function | 0.1 | | 0.1 | 0 | 0 | 0 | 0 |
| **4+*4.013* | No function | 0 | | 0 | 0.7 | 0 | 0 | 0 |
| **5* | No function | 8.1 | 5.2 | 7 | 2.4 | 2.2 | 2.5 | 3.5 |
| **6* | No function | 0 | 0 | 0.3 | 2.1 | 0.3 | 0.1 | 0 |
| **7* | No function | 0 | 0 | 0 | 0 | 0 | 0.9 | 0 |
| **11* | No function | 0 | | 0.3 | 0 | 0 | 0 | 0 |
| **12* | No function | 0.2 | 0.3 | 0 | 0 | 0 | 0 | 0 |
| **13* | No function | 0.1 | | 0.3 | 0.2 | 0.1 | 0.1 | 0 |
| **15* | No function | 0.2 | 0.6 | 0 | 0 | 0 | 0 | 0 |
| **20* | No function | 0 | | 0 | 0 | 0 | 0 | 0.1 |
| **21* | No function | 0 | 0 | 0 | 0 | 0 | 0 | 0.5 |

| | | Allele frequencies (%) | | | | | | |
|---|---|---|---|---|---|---|---|---|
| *CYP2D6* allele | CPIC function | SSA in this study (n=947) | Previous SSA studies[a] (n=2248) | African American/ Afro-Caribbean (n=157) | EUR (n=498) | AMR (n=345) | SAS (n=481) | EAS (n=502) |
| **31* | No function | 0 | | 0 | 0.2 | 0.6 | 0 | 0 |
| **36* | No function | 0.4 | | 0.3 | 0 | 0 | 0 | 0.2 |
| **36x2* | No function | 0 | | 0 | 0 | 0 | 0 | 0.3 |
| **40* | No function | 1.4 | 1.3 | 0.3 | 0 | 0 | 0 | 0 |
| **42* | No function | 0.1 | | 0 | 0 | 0 | 0 | 0 |
| **56* | No function | 0.2 | | 0 | 0 | 0 | 0 | 0 |
| **69* | No function | 0.1 | | 0 | 0.1 | 0 | 0.2 | 0.3 |
| **68* | No function | 0 | | 0 | 0 | 0.1 | 0.1 | 0 |
| **68+*4* | No function | 0.1 | | 1 | 5.7 | 2.6 | 2.2 | 0 |
| **68x2+*4* | No function | 0 | | 0 | 0 | 0.3 | 0 | 0 |
| **99* | No function | 0 | | 0 | 0 | 0 | 0.2 | 0 |
| **1x2* | Increased | 0.4 | 1.1 | 0.3 | 0.5 | 1.2 | 0.6 | 0.3 |
| **1x3* | Increased | 0.2 | | 0 | 0 | 0.1 | 0 | 0 |
| **1x2+*83* | Increased | 0 | | 0 | 0 | 0 | 0.1 | 0 |
| **2x2* | Increased | 1.9 | 1.7 | 1.3 | 1.5 | 0.6 | 0.4 | 0.5 |
| **2x3* | Increased | 0.1 | | 0 | 0.1 | 0 | 0 | 0.1 |
| **2x4* | Increased | 0.2 | | 0 | 0 | 0 | 0 | 0 |
| **35x2* | Increased | 0 | 0 | 0 | 0 | 0.1 | 0 | 0 |
| **45x2* | Increased | 0.1 | | 0 | 0 | 0 | 0 | 0 |
| **45x3* | Increased | 0.1 | | 0 | 0 | 0 | 0 | 0 |
| **1+*90* | Uncertain | 0 | | 0 | 0 | 0 | 0 | 0.1 |
| **22* | Uncertain | 0 | | 0 | 0.3 | 0 | 0 | 0 |
| **28* | Uncertain | 0 | 0 | 0 | 0.5 | 0.1 | 0 | 0 |
| **32* | Uncertain | 0 | 0.3 | 0 | 0.3 | 0 | 0.2 | 0 |
| **43* | Uncertain | 0.8 | 1.7 | 1 | 0.1 | 0 | 1 | 0 |

| | | Allele frequencies (%) | | | | | | |
|---|---|---|---|---|---|---|---|---|
| **_CYP2D6_ allele** | **CPIC function** | **SSA in this study (n=947)** | **Previous SSA studies[a] (n=2248)** | **African American/ Afro-Caribbean (n=157)** | **EUR (n=498)** | **AMR (n=345)** | **SAS (n=481)** | **EAS (n=502)** |
| **43x2* | Uncertain | 0.1 | | 0 | 0 | 0.1 | 0 | 0 |
| **52* | Uncertain | 0 | 0.8 | 0 | 0 | 0 | 0 | 0.1 |
| **71* | Uncertain | 0 | | 0 | 0 | 0 | 0 | 0.6 |
| **73* | Unknown | 0.2 | 0.5 | 0 | 0 | 0 | 0 | 0 |
| **74* | Unknown | 0.1 | 0.5 | 0 | 0 | 0 | 0 | 0 |
| **82* | Unknown | 0 | | 0 | 0 | 0.4 | 0 | 0 |
| **86* | Unknown | 0 | | 0 | 0 | 0 | 2.3 | 0 |
| **106* | Unknown | 1.5 | | 0 | 0 | 0.1 | 0 | 0 |
| **108* | Unknown | 0 | | 0 | 0.3 | 0 | 0 | 0 |
| **111* | Unknown | 0 | | 0 | 0.4 | 0 | 0.8 | 0 |
| **112* | Unknown | 0 | | 0 | 0 | 0 | 0.2 | 0 |
| **113* | Unknown | 0 | | 0 | 0 | 0 | 0.8 | 0 |
| **117* | Unknown | 0 | | 0 | 0.4 | 0 | 0 | 0 |
| **119* | Unknown | 0 | | 0 | 0 | 0 | 0 | 0.1 |
| **121* | Unknown | 0 | | 0.3 | 0 | 0 | 0 | 0 |
| **122* | Unknown | 0.1 | | 0.3 | 0 | 0 | 0 | 0 |
| **125* | Unknown | 0.3 | | 0.3 | 0 | 0 | 0 | 0 |
| **139* | Unknown | 0.1 | | 0 | 0 | 0 | 0.1 | 0 |
| **144* | Unknown | 0 | | 0 | 0 | 0 | 0 | 0.1 |
| **149* | Unknown | 0.3 | | 0 | 0 | 0 | 0 | 0.1 |

n, individuals; SSA; sub-Saharan African participants; EUR, European participants; SAS, South Asian participants; EAS, East Asian participants; CPIC, Clinical Pharmacogenetics Implementation Consortium; PharmGKB, Pharmacogenomics Knowledge Base; [a] See PharmGKB and CPIC *CYP2D6* reference materials (https://www.pharmgkb.org/page/cyp2d6RefMaterials)

**Table S4**: Unresolved *CYP2D6* diplotypes with potential novel alleles in sub-Saharan African populations

| # | Background alleles | Additional core variant(s) | Variant type | Number of participants | Country/dataset [a] |
|---|---|---|---|---|---|
| 1 | *1/*43 | rs61731586~8330G>A | Missense (R380H) | 1 | Nigeria (1000G) |
| 2 | *2/*4 | rs61736512 6679G>A; possible CYP2D6 duplication | Missense (V136M) | 1 | Sierra Leone (1000G) |
| 3 | *2/*17 | rs778690377~6968C>T | Missense (L203F) | 1 | Kenya (1000G) |
| 4 | *2/*17 | rs373243894~5141C>T | Missense (P41L) | 1 | Kenya (1000G) |
| 5 | *2/*29 | rs556882139~6879G>A | Missense (R173H) | 1 | Gambia (1000G) |
| 6 | *2/*139 | rs566108360~8209G>C + rs1135829~7838A>G; possible CYP2D6 duplication | Missense (G340R, N285S) | 1 | Congo (SGDP) |
| 7 | *17/*29 | rs532668079~9065G>A | Missense (R441H) | 1 | Nigeria (1000G) |
| 8 | *1/*2 | *4, *17, and *29-defining variants in the same sample | | 1 | South Africa |
| 9 | *1/*2 | Possible *68-like CYP2D6/2D7 hybrid (lacking 5119C>T) | Structural variant | 1 | South Africa |
| 10 | *1/*2 | Duplicated CYP2D6 haplotype (*1 + rs565013903~7600G>A) | Structural variant | 1 | Namibia (SGDP) |
| 11 | *45/*46 | Possible *68-like CYP2D6/2D7 hybrid (lacking 5119C>T) | Structural variant | 1 | Gambia (1000G) |
| 12 | *29/*45 | Possible *68-like CYP2D6/2D7 hybrid (lacking 5119C>T) | Structural variant | 1 | Kenya (1000G) |
| 13 | *45/*45 | Possible *68-like CYP2D6/2D7 hybrid (lacking 5119C>T) | Structural variant | 1 | Kenya (1000G) |

SGDP, Simons Genome Diversity Project; 1000G, 1000 Genomes Project; [a] IDs for Coriell and SGDP samples are provided in Table S5 as these samples can potentially be used as reference materials.

**Figure S4**: Examples of novel and confirmatory *CYP2D6* suballeles in SSA characterised via XL-PCR and HiFi sequencing

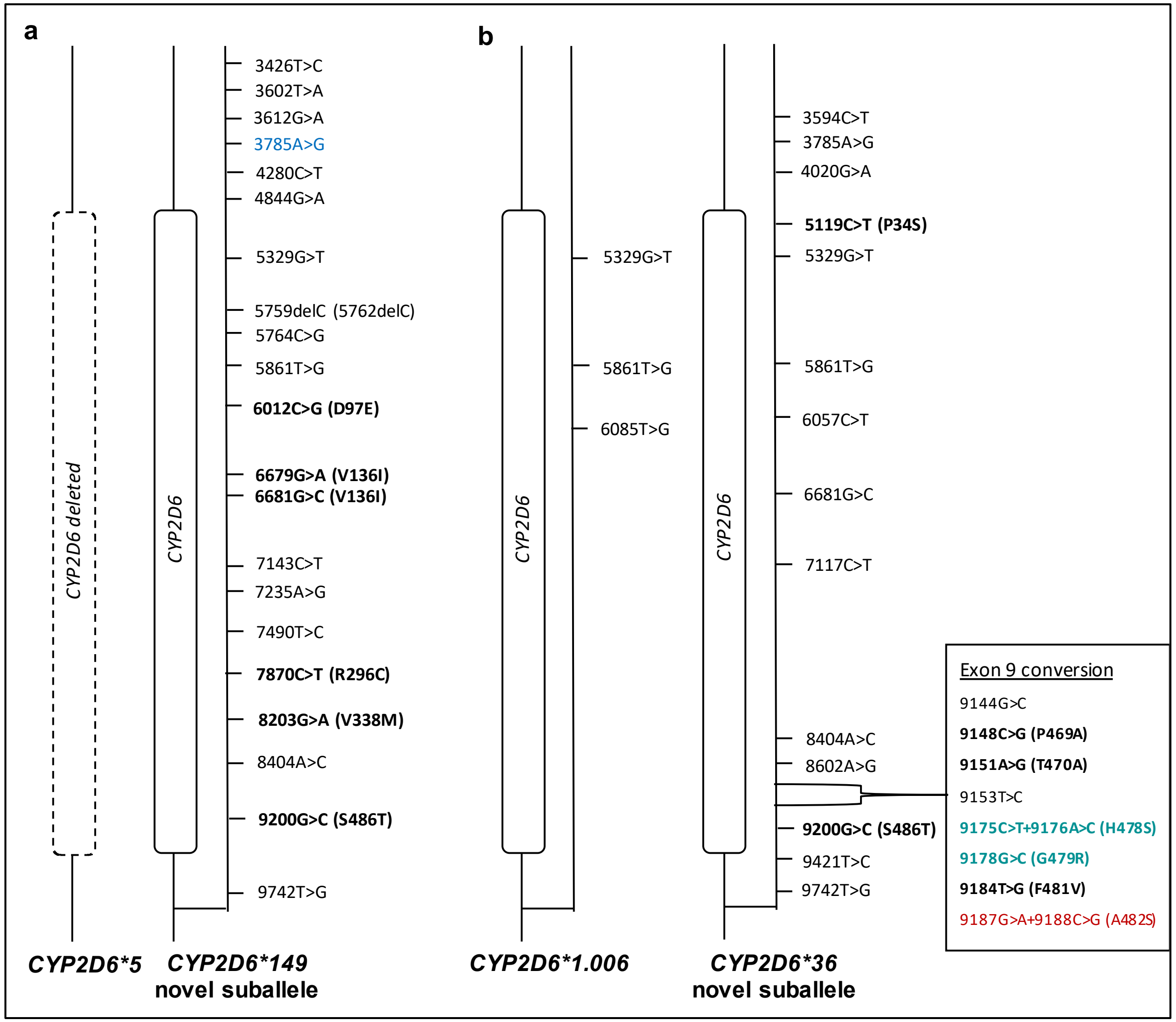


Panel (**a**) shows a novel *CYP2D6*149* suballele from this study. *CYP2D6*149* is defined by rs76802407 (D97E) in phase with the **29*-defining variants, and it has recently been assigned a novel major star allele status (with definitive evidence) by PharmVar (https://www.pharmvar.org). The allele depicted here differs from *CYP2D6*149.001* as it has the NG_008376.4:g.3785A>G variant. Panel (**b**) shows a novel *CYP2B6*36* suballele called in a participant from Burkina Faso. HiFi data facilitated the detection of the exon 9 conversion SNVs. However, some of these SNVs were not called by the variant detection tool DeepVariant possibly due to lack of sufficient read support. The SNVs indicated in teal were observed during manual inspection in the Integrative Genomics Viewer (IGV) while there were virtually no reads supporting the SNVs indicated in red text. These haplotypes are currently under review by PharmVar and could instead be integrated as confirmatory suballeles if appropriate.

**Table S5**: Sample information for participants with publicly available high coverage WGS data containing potential novel *CYP2D6* star alleles. The Coriell DNA samples from these participants could potentially be added to GeT-RM reference materials for *CYP2D6* genotyping in pharmacogenomics testing laboratories.

| **Coriell/SGDP IDs** | **Predicted Consensus Diplotype** | **Country/ population** | **Primary project** |
|---|---|---|---|
| HG02570 | *1/[*1+rs140900383~7471C>T (A226V)] | GWD | 1000G |
| LP6005441-DNA_A11 | [*2/*1 + rs565013903~7600G>A (R269P)]; (cn=3) | Namibia | SGDP |
| LP6005443-DNA_G08 | [*1+rs565013903~7600G>A]/[*1+rs565013903~7600G>A] | Namibia | SGDP |
| HG03428 | *5/[*1+rs567606867~7004G>A (E215K)] | MSL | 1000G |
| NA19468 | *1/[*2+rs368858603~7609_7610insA (fs)] | LWK | 1000G |
| SS6004471 | *2/[*2 + rs368858603~7609_7610insA (fs) + rs374616348~6628G>T (V119L)] | Congo | SGDP |
| NA19314 | *5/[*2 + rs28371704~6002A>G (H94R) + rs28371703~5992C>A (L91M)] | LWK | 1000G |
| HG03559 | *2/[*2+rs376636053~6655T>C (W128R)] | MSL | 1000G |
| HG03469 | *2/[*2+rs769157652~8873G>A (E410K)] | MSL | 1000G |
| LP6005592-DNA_C05 | *5/[*17+rs1450231864~8231T>C (M347T)] | South Africa | SGDP |
| HG02666 | *1/[*29 + rs76802407~6012C>G (D97E)] | GWD | 1000G |
| HG02870 | *1/[*29+rs76802407~6012C>G (D97E)] | GWD | 1000G |
| HG03442 | *2x2/[*29 + rs76802407~6012C>G (D97E)] | MSL | 1000G |
| NA18933 | *5/[*29+rs201006451~6767C>T (A165V)] | YRI | 1000G |
| HG02840 | *17/[*29+rs536109057~5173C>T (stop-gained)] | GWD | 1000G |
| HG02860 | *2/[*29+rs536109057~5173C>T (stop-gained)] | GWD | 1000G |
| NA19130 | *106/[*29+rs760940331~9096G>A (M451I)] | YRI | 1000G |
| HG02807 | *17/[*29+rs760940331~9096G>A (M451I)] | GWD | 1000G |
| NA19316 | *2/[*41 + rs141824015~8206A>C (I339L)] | LWK | 1000G |
| HG02623 | *2/[*45 + rs3915951~8177G>T (R329L)] | GWD | 1000G |
| HG02642 | *17/[*45 + rs3915951~8177G>T (R329L)] | GWD | 1000G |
| NA19222 | [*1/*43] + rs61731586~8330G>A (R380H) | YRI | 1000G |
| HG03470 | [*2/*4] + rs61736512~6679G>A (V136M); cn=3 | MSL | 1000G |
| NA19472 | [*2/*17] + rs778690377~6968C>T (L203F) | LWK | 1000G |
| NA19026 | [*2/*17] + rs373243894~5141C>T (P41L) | LWK | 1000G |
| HG02852 | [*2/*29] + rs556882139~6879G>A (R173H) | GWD | 1000G |
| LP6005441-DNA_A08 | [*2/*139] + rs566108360~8209G>C (G340R) + rs1135829~7838A>G (N285S) | Congo | SGDP |
| HG03313 | [*17/*29] + rs532668079~9065G>A (R441H) | ESN | 1000G |
| HG02621 | [*45/*46] + Possible *68-like *CYP2D6/2D7* hybrid (lacking 5119C>T) | GWD | 1000G |
| NA19017 | [*29/*45] + Possible *68-like *CYP2D6/2D7* hybrid (lacking 5119C>T) | LWK | 1000G |
| NA19456 | [*45/*45] + Possible *68-like *CYP2D6/2D7* hybrid (lacking 5119C>T) | LWK | 1000G |

cn, copy number; fs, frameshift; 1000G, 1000 Human Genomes Project; SGDP, Simons Genome Diversity Project; ESN, Esan in Nigeria; LWK, Luhya in Webuye Kenya; GWD, Gambian from Western Divisions (Mandinka); MSL, Mende in Sierra Leone; YRI, Yoruba in Ibadan, Nigeria; GeT-RM, Genetic Testing Reference Materials Coordination Program.

**Table S6**: VEP plugin predictions of the deleteriousness of core variants defining potential novel African ancestry *CYP2D6* star alleles identified in the study.

| Core variants | Consequence | CADD | SIFT/ SIFT Indel | Polyphen-2 | LRT | PROVEAN | VEST | LOFTEE | Consensus |
|---|---|---|---|---|---|---|---|---|---|
| rs140900383 | Missense (A226V) | | | | | | | | |
| rs141756339 | Missense (R474Q) | | | | | | | | |
| rs565013903 | Missense (R269P) | X | X | X | X | X | X | | X |
| rs567606867 | Missense (E215K) | | | | | | X | | |
| rs368858603 | Frameshift (T272TX) | | X | | | | | X | X |
| rs374616348 | Missense (V119L) | | | | | | | | |
| rs28371704 | Missense (H94R) | | | | | X | | | |
| rs28371703 | Missense (L91M) | X | X | X | X | | | | X |
| rs375715419 | Missense (T458A) | X | X | | X | X | | | X |
| rs376636053 | Missense (W128R) | X | X | X | | X | X | | X |
| rs769157652 | Missense (E410K) | X | | | | | | | |
| rs747089665 | Missense (R414C) | X | | | | X | | | |
| rs1450231864 | Missense (M347T) | X | | X | X | X | X | | X |
| rs201006451 | Missense (A165V) | | | | | | X | | |
| rs536109057 | Stop-gained (Q52*) | | | | | | | X | |
| rs760940331 | Missense (M451I) | X | X | X | X | X | X | | X |
| rs141824015 | Missense (I339L) | | | X | X | | X | | X |
| rs3915951 | Missense (R329L) | X | X | | | | | | |
| rs61731586 | Missense (R380H) | X | | | X | X | X | | X |
| rs61736512 | Missense (V136I) | | | | | | | | |
| rs778690377 | Missense (L203F) | | | X | | | | | |
| rs373243894 | Missense (P41L) | X | X | X | X | X | X | | X |
| rs556882139 | Missense (R173H) | X | X | | | | | | |
| rs532668079 | Missense (R441H) | X | X | X | X | X | X | | X |
| rs566108360 | Missense (G340R) | X | X | X | X | X | X | | X |
| rs1135829 | Missense (N285S) | | | | | X | | | |

X, indicates that the variant was predicted to be deleterious by the corresponding plugin; CADD, Combined Annotation Dependent Depletion; SIFT, Sorting Intolerant from Tolerant; Polyphen-2, Polymorphism Phenotyping (version 2); LRT, Likelihood Ratio Test; PROVEAN, Protein Variation Effect Analyzer; VEST, Variant Effect Scoring Tool (version 4); LOFTEE, Loss-Of-Function Transcript Effect Estimator. For each plugin a variant was considered deleterious if the scores met the following ADME-optimised thresholds suggested by Zhou et al. (2017): CADD PHRED (>19.19), SIFT (<0.0376), Polyphen-2 (>0.3841), LRT (<0.0025), PROVEAN (<-3.28), VEST4 (>0.4534). The consensus indicates a deleteriousness prediction where at least half the plugins used to assess the variant predicted it to be deleterious.

## References:

Zhou, Y., Mkrtchian, S., Kumondai, M., Hiratsuka, M. and Lauschke, V. M. (2019). An optimized prediction framework to assess the functional impact of pharmacogenetic variants. *Pharmacogenomics J* 19, 115–126.

**Table S7**: Potential novel *CYP2D6* star alleles identified in participants from global biogeographical groups included for comparison in the study. The Coriell DNA samples from these participants could potentially be added to GeT-RM reference materials for *CYP2D6* genotyping in pharmacogenomics testing laboratories.

| Coriell IDs | Predicted Consensus Diplotype | Population | Primary project |
|---|---|---|---|
| HG02318 | *1/[*1+rs575708064~8332G>A (D381N)] | ACB | 1000G |
| HG01491, HG01372, HG01149, HG01072<br>HG01086<br>HG01363 | *1/[*1 + rs538707090~6935G>A (G192R)]<br>*31/[*1 + rs538707090~6935G>A (G192R)]<br>*2/[*1 + rs538707090~6935G>A (G192R)] | AMR | 1000G |
| HG01139, HG01341<br>HG01551<br>HG01133 | *4/[*2 + rs28371696~5096G>A (R26H)]<br>*5/[*2 + rs28371696~5096G>A (R26H)]<br>*2/[*2 + rs28371696~5096G>A (R26H)] | AMR | 1000G |
| HG01565 | *1/[*1 + rs563185985~6058G>A (G113R)] | AMR | 1000G |
| HG01992 | *1/[*1 + rs534682262~7609C>T (T272I)] | AMR | 1000G |
| NA19750 | *1/[*1 + rs1135828~7630T>A (M279K)] | AMR | 1000G |
| HG01101 | *41/[*2 + rs538036869~8916A>C (Q424P)] | AMR | 1000G |
| HG01097 | *31/[*13 + (*2 + rs78762568~8867G>A; V408I)] | AMR | 1000G |
| HG01257 | *2/[*2 + rs565444796~5909G>A (R63H)] | AMR | 1000G |
| HG01403 | [*1/*2] + rs544790460~7461G>T (V223L) | AMR | 1000G |
| HG01083 | [*1/*2] + rs376636053~6655T>C (W128R) | AMR | 1000G |
| NA20320 | *5/[*2 + rs146819268~7518C>T (R242C)] | ASW | 1000G |
| NA20340 | *29/[*29 + rs76802407~6012C>G (D97E)] now *CYP2D6*149* | ASW | 1000G |
| HG02384, HG00693, NA18559 | *1/[*1 + rs769258~5050G>A (V11M)] | EAS | 1000G |
| NA19074 | *5/[*10 + rs3915951~8177G>T (R329L)] | EAS | 1000G |
| HG02379 | *36+10/[*1 + rs567606867~7004G>A (E215K)] | EAS | 1000G |
| HG01600, HG02121 | *36x2+*10/[*10 + rs774778807~6907_6909delGAG (S183del)] | EAS | 1000G |
| NA18641 | *36+*10/[*1 + rs528725654~6776C>A (S168Y)] | EAS | 1000G |

| | | | |
|---|---|---|---|
| HG00542 | *36+*10/[*1 + rs770277909~6725A>G (Q151R)] | EAS | 1000G |
| HG02026 | *36+*10/[*10 + new_rsID~8823delCAAGGGA] | EAS | 1000G |
| NA18630 | *36+*10/[*2 + rs1135822~6631T>A (F120I) + rs1135823~6637G>T (A122S)] | EAS | 1000G |
| HG00566 | *36+*10/[*2 + rs377591409~6638C>T (A122V)] | EAS | 1000G |
| NA18942 | *2/[*2 + rs78762568~8867G>A (V408I)] | EAS | 1000G |
| HG02187 | *10/[*1 + rs140513104~7958C>T (P325L)] | EAS | 1000G |
| NA19682 | *10/[*1 + rs28371703~5992C>A (L91M) + rs28371704~6002A>G (H94R)] | EAS | 1000G |
| HG00595 | [*10/*36x2+*83+*10] (unresolved diplotype) | EAS | 1000G |
| HG00458 | [*1/*36xN+*10xN] – unresolved diplotype | EAS | 1000G |
| HG00346 | *1/[*1 + rs199722016~9212A>G (T490C)] | EUR | 1000G |
| NA12872 | *9/[*35 + rs1058172~8285G>A (R365H)] | EUR | 1000G |
| NA12776 | *1x2/[*1 + rs200234159~7946T>C (M321T)] | EUR | 1000G |
| NA20815 | [*41/*9] + rs3915951~8177G>T (R329L) | EUR | 1000G |
| NA11919 | [*10/*2] + rs371793722~7017T>C (F219S) | EUR | 1000G |
| NA20798 | [*1/*41] + rs141289473~7942C>T (L320F) | EUR | 1000G |
| NA11830 | [*1/*4] + rs138229048~9129G>C (Q462H) | EUR | 1000G |
| HG01785 | [*1/*2] + rs150216909~7969C>T (R329C) | EUR | 1000G |
| HG03941 | *1/[*1 + rs566833518~9227T>C (V495A)] | SAS | 1000G |
| HG03709 | *1/[*1 + rs3021084~8934C>T (P430L)] | SAS | 1000G |
| HG02774 | *86/[*2 + rs777691989~7480delTCC (L231del)] | SAS | 1000G |
| HG03870 | *41/[*41 + rs565013903~7600G>A (R269Q)] | SAS | 1000G |
| HG04214 | *2/[*1 + rs267608295~8218C>T (R343W)] | SAS | 1000G |
| HG03803 | *13/[*1 + rs542114265~8232G>A (M347I)] | SAS | 1000G |
| HG04182 | [*2/*41] + rs149686350~8298C>G (I369M) | SAS | 1000G |
| HG03898 | [*2/*41] + rs1135823~6637G>T (A122S) | SAS | 1000G |
| HG03772 | [*2/*4] + rs567340138~7840C>A (P286T) | SAS | 1000G |

| | | | |
|---|---|---|---|
| HG04014 | [*2/*4] + rs558523758~7563G>C (E257Q) | SAS | 1000G |
| HG03593 | [*1/*41] + rs1135830~7916C>T (stop-gained) | SAS | 1000G |
| HG03800 | [*1/*2] + rs544811063~8324C>T (T378I) | SAS | 1000G |
| HG04194 | [*1/*2] + rs531010318~7013G>C (G218R) | SAS | 1000G |
| HG03802 | [*1/*10] + rs556882139~6879G>A (R173H) | SAS | 1000G |
| HG04002 | *2/(*1x2+*83) – unresolved diplotype | SAS | 1000G |
| HG02778, NA07347 | *1/(*68-like + *2); has intron 1 breakpoint; lacks rs1065852 | SAS, EUR | 1000G |

cn, copy number; fs, frameshift; 1000G, 1000 Human Genomes Project; GeT-RM, Genetic Testing Reference Materials Coordination Program; ACB, African Caribbean in Barbados; ASW, People with African Ancestry in Southwest USA; EUR, European participants; AMR, Admixed American participants; EAS, East Asian participants; SAS, South Asian participants.
